# Provitamin A Carotenoids in Grain Reduce Aflatoxin Contamination of Maize While Combating Vitamin A Deficiency

**DOI:** 10.3389/fpls.2019.00030

**Published:** 2019-01-29

**Authors:** Willy B. Suwarno, Pattama Hannok, Natalia Palacios-Rojas, Gary Windham, José Crossa, Kevin V. Pixley

**Affiliations:** ^1^International Maize and Wheat Improvement Center, Texcoco, Mexico; ^2^Department of Agronomy and Horticulture, Faculty of Agriculture, Bogor Agricultural University, Bogor, Indonesia; ^3^Department of Agronomy, University of Wisconsin-Madison, Madison, WI, United States; ^4^Corn Host Plant Resistance Research Unit, United States Department of Agriculture-Agricultural Research Service, Starkville, MS, United States

**Keywords:** aflatoxin, beta-carotene, beta-cryptoxanthin, biofortification, maize breeding, mycotoxins, vitamin A deficiency

## Abstract

Aflatoxin contamination of maize grain and products causes serious health problems for consumers worldwide, and especially in low- and middle-income countries where monitoring and safety standards are inconsistently implemented. Vitamin A deficiency (VAD) also compromises the health of millions of maize consumers in several regions of the world including large parts of sub-Saharan Africa. We investigated whether provitamin A (proVA) enriched maize can simultaneously contribute to alleviate both of these health concerns. We studied aflatoxin accumulation in grain of 120 maize hybrids formed by crossing 3 *Aspergillus flavus* resistant and three susceptible lines with 20 orange maize lines with low to high carotenoids concentrations. The hybrids were grown in replicated, artificially-inoculated field trials at five environments. Grain of hybrids with larger concentrations of beta-carotene (BC), beta-cryptoxanthin (BCX) and total proVA had significantly less aflatoxin contamination than hybrids with lower carotenoids concentrations. Aflatoxin contamination had negative genetic correlation with BCX (-0.28, *p* < 0.01), BC (-0.18, *p* < 0.05), and proVA (-0.23, *p* < 0.05). The relative ease of breeding for increased proVA carotenoid concentrations as compared to breeding for aflatoxin resistance in maize suggests using the former as a component of strategies to combat aflatoxin contamination problems for maize. Our findings indicate that proVA enriched maize can be particularly beneficial where the health burdens of exposure to aflatoxin and prevalence of VAD converge with high rates of maize consumption.

## Introduction

Aflatoxin contamination of maize is a serious health threat and burden for millions of maize consumers worldwide. Aflatoxin is a secondary metabolite produced by the ubiquitous *Aspergillus flavus* fungus, and is very toxic to humans and animals. Consumption of aflatoxin contaminated food is particularly serious for children because it leads to compromised immune system and increased morbidity and mortality from malaria and other diseases, reduced efficiency of use for various macro- and micro-nutrients, and stunting or underweight development (Williams et al., 2004; Wild, 2007). In adults, aflatoxin is mainly associated with liver and other cancers, but chronic exposure to aflatoxin has also been associated with increased occurrence of micronutrient deficiencies and increased burden of diseases (e.g., malaria and HIV/AIDS) from weakened immune system have also been reported or postulated (Williams et al., 2004). Exposure to unsafe levels of aflatoxin in maize and maize products is common for large populations in sub-Saharan Africa, resulting in chronic morbidity and events of multiple deaths from aflatoxicosis (Williams et al., 2004; Wild, 2007; Misihairabgwi et al., 2017; Mahuku et al., 2019). Although we focus on human health concerns of aflatoxin contamination in maize, aflatoxin in grains other than maize, and in grains used in animal feeds are also of huge economic and health concern.

Vitamin A deficiency (VAD) also affects millions of maize consumers, particular children and pregnant women, and especially in sub-Saharan Africa and Southeast Asia. Biofortification, or breeding of provitamin A (proVA) enriched maize varieties is ongoing at CIMMYT and other HarvestPlus partner institutions (Pixley et al., 2013; Tanumihardjo et al., 2017). Several proVA biofortified maize varieties have been released in sub-Saharan Africa, where efficacy trials have demonstrated their potential to benefit maize consuming, VAD populations (Gannon et al., 2014). In contrast to the rapid success of proVA breeding efforts, progress in breeding maize varieties with resistance to *A. flavus* infection and aflatoxin contamination has proven difficult and elusive (Henry et al., 2013; Warburton and Williams, 2014). Bhatnagar-Mathur et al. (2015) reviewed various methods to reduce aflatoxin contamination of grain, including plant breeding, biological control in the field and post-harvest handling of grain (see also Gressel and Polturak, 2018).

There is considerable evidence suggesting the potential of breeding maize with enhanced concentrations of carotenoids to have favorable health benefits for reducing the burden of aflatoxin contamination of maize grain while also alleviating VAD. Consumption of carotenoids, specifically beta-carotene (BC) or beta-cryptoxanthin (BCX), has been associated with reduced risk and decreased morbidity for diverse types of human cancer, including lung, oral, pharynx, larynx, esophagus, colon, prostate, and liver (Gradelet et al., 1998; Fiedor and Burda, 2014). Krinsky (1991) cited 13 studies in mouse, 8 in hamster, and 5 in rat model for which BC inhibited tumor development and or growth. The precise modes by which carotenoids exercise anti-carcinogenic effects are not fully understood, but several contributing mechanisms have been described. It is important to note that the specific carotenoids, and not vitamin A (retinol), have these beneficial effects (Krinsky, 1993; Alpsoy et al., 2009).

Preston and Williams (2005) explained that aflatoxin B1 (AFB1) is bio-converted to its more damaging form, AFBE, by the action of cytochrome genes (e.g., *CYP1A*). AFBE then binds DNA at a specific codon within the *TP53* tumor-suppressing gene, mutating it to inactivate its cancer-protective actions (Sporn et al., 1966; Scaife, 1971; Preston and Williams, 2005). BC acts in several ways to counter these carcinogenic effects of aflatoxin: (1) BC up-regulates expression of *TP53*, thereby competing with AFBE’s strategy to reduce production of *TP53* transcript (Reddy et al., 2006), (2) BC acts on *CPY1A* resulting in decreased production of AFBE and increased metabolism of AFB1 to aflatoxin M1, a less toxic metabolite (Krinsky, 1993; Gradelet et al., 1997, 1998), and (3) BC reduces the production of AFB1 through its antioxidant activities (Krinsky, 1989; Ponts et al., 2006; Fiedor and Burda, 2014; Montibus et al., 2015).

Montibus et al. (2015) reviewed the importance of antioxidants in down-regulating secondary metabolism and production of mycotoxins, including AFB1, by fungi. They described how fungal invasion of plant cells commonly induces a defensive “oxidative burst,” or release of reactive oxygen species (ROS), intended to cause a hypersensitive, cell death reaction to limit spread of the fungus. Some fungi, including *A. flavus* and *Fusarium* spp. have evolved oxidation-demanding secondary metabolism pathways that quench ROS while and by producing mycotoxins (e.g., AFB1 by *A. flavus*, and deoxynivalenol (DON) by *Fusarium spp.*). Carotenoids, especially BC and BCX, are highly effective antioxidants that quench some of the ROS produced in response to *A. flavus* invasion and thereby reduce aflatoxin production. Many publications describe the antioxidant role of flavonoids in down-regulating production of fumonisins and DON (e.g., Boutigny et al., 2009; Atanasova-Penichon et al., 2016; Giordano et al., 2017), and some allude to, or specifically mention the analogous nature of carotenoids in combating aflatoxin production.

Norton (1997) reported that carotenoid compounds (obtained from commercial laboratories) that occur in yellow maize, including BC, BCX, ZX, and LT, significantly reduced aflatoxin production by *A. flavus in vitro*. Another *in vitro* study found that commercially-obtained BC inhibited aflatoxin biosynthesis by >70% for 38 *Aspergillus* genotypes isolated from Illinois maize (Wicklow et al., 1998). More recently, Bhatnagar-Mathur et al. (2015) discussed possibilities, albeit unrelated to carotenoid concentrations in grain, to apply transgenic, RNAi and gene editing approaches to combat aflatoxin production. Subsequently, Thakare et al. (2017) demonstrated that transgenic, host-induced gene silencing (HIGS) of the *aflC* gene, which encodes an enzyme in the *Aspergillus* aflatoxin biosynthetic pathway, inhibited aflatoxin biosynthesis by *Aspergillus* in maize kernels. Although they did not evaluate *Aspergillus* and aflatoxin, Díaz-Gómez et al. (2016) reported that transgenic maize lines engineered to contain moderate concentrations of BC (5.9 μg g^-1^) and BCX (3.7 μg g^-1^) had lower levels of fumonisin accumulation (generally produced by *Fusarium verticillioides* and *F. proliferatum* fungi) than their non-transgenic, white-grained counterparts.

There are no published reports about the relationship between carotenoids content and aflatoxin accumulation in maize grain. Therefore, our objective was to investigate the hypothesis that proVA biofortified maize can have an additional health benefit by reducing aflatoxin contamination of maize grain. Specifically, we investigated the relationship between carotenoids concentrations and aflatoxin accumulation in grain of *A. flavus* resistant or susceptible hybrids with contrasting concentrations of BC, BCX, ZX, and LT.

## Materials and Methods

### Germplasm Materials

Twenty CIMMYT orange or yellow maize lines were chosen based on their high (lines L1–L10) or low (L11–L20) total provitamin A (proVA) concentrations (Supplementary Table S1). The high proVA lines were promising lines within CIMMYT’s proVA biofortification breeding program, while the low proVA lines were also advanced lines, but would be discarded due to their low proVA concentrations. Six white maize inbred lines were chosen for use as testers based on prior information about their resistance (lines R1–R3) or susceptibility (S1–S3) to *Aspergillus* ear rot and aflatoxin accumulation (personal communication, George Mahuku, former CIMMYT maize pathologist) (Supplementary Table S2). We used white tester lines because there were no orange or yellow resistant lines available. The 20 lines were used as females for crosses with the six testers as males, resulting in 120 F_1_ hybrids.

### Field Experiments

The 120 F_1_ hybrids were evaluated in five environments: Agua Fria, Puebla, Mexico (AF) (20°32′N, 97°28′W) and Tlaltizapan, Morelos, Mexico (TL) (18°41N, 99°07W) during 2012 and 2013, and Mississippi State University, Starkville, Mississippi, United States (MS) (33°28′N, 88°46′W) during 2012. The experimental design was an alpha 0,1 lattice (Patterson et al., 1978) with four replications (AF and TL) or three replications (MS). Plots were 2 m long with 10 plants and between-row spacing of 0.75 m. All plants were artificially inoculated with *A. flavus* as described below. All primary ears from each plot were hand-harvested, visually scored for *Aspergillus* ear rot symptoms, as described below, and collected as a bulk. The harvested maize ears were air-dried for a week to reduce grain moisture to 13% prior to shelling for laboratory analyses of F2 grains. Supplementary Table S3 presents the planting, inoculation and harvest months, as well as average daily high and low temperatures, rainfall and percent humidity for the trials.

An additional un-replicated set of the F_1_ hybrids was grown as a nursery at each location (except MS) to enable carotenoids quantification. These nurseries were non-inoculated and plants were self-pollinated by hand. Carotenoid concentrations of F2 grain from these nurseries were measured at CIMMYT’s “Evangelina Villegas” Maize Quality Laboratory immediately after harvest.

### Field Inoculation With *Aspergillus flavus*

The *A. flavus* strains used for inoculum, final concentrations of inoculum, number of inoculation points on the maize ears and volume of conidial suspensions differed between the Mexican and United States sites based on prior research experience at each site and need to use strains endemic for each site, i.e., not to introduce new strains or use strains that might not be adapted to conditions at any site.

*Aspergillus* inoculation of the four trials in Mexico followed CIMMYT’s standard protocols (Drepper and Renfro, 1990). Four isolates of toxigenic *A. flavus* were grown in separate jars containing sterilized maize grain for 2 weeks at 25°C and subsequently kept at 4°C until use. Conidia were then collected by adding sterilized tween 20-water into the jars, vigorously hand-shaking and filtering the inoculum. Spores were counted using a haemocytometer and diluted to achieve a final concentration of 10^6^ conidia ml^-1^. Maize ears were inoculated 14–18 days after silking, with the mixed inoculum of 4 isolates of *A. flavus*. Using a needle inoculation technique (Drepper and Renfro, 1990), the primary ear on each maize plant was injected with the mixed inoculum at two positions, i.e., on the side and on the tip of the ear.

The inoculation procedure at MS was instructed by the CHPRRU (Zummo and Scott, 1989). Briefly, *A. flavus* isolate NRRL 3357 was increased in flasks containing 50 g of sterile maize cob grits (size 2040, Grit-O-Cob, The Andersons Co., Maumee, OH, United States) and 100 ml of sterile distilled water, and incubated at 28°C for 21 days. Conidia were collected from grits by adding sterilized tween 20-water and filtering through layered sterile cheesecloth. The concentration of conidia was counted with a haemocytometer and diluted to obtain 9 × 10^7^ conidia ml^-1^. The primary ear of each plant was inoculated 7 days after silking using the side-needle technique with 3.4 ml of the conidial suspension (Zummo and Scott, 1989; Williams et al., 2013).

### Visual Evaluations of *Aspergillus* Ear Rot and Aflatoxin Contamination

The visible fungal colonization, or *Aspergillus* ear rot (ER) symptom scores, were assessed for ears at harvest using a scale of 1–5, where 1 is 0% and 5 is 100% of visible fungal infection (adapted from Campbell and White, 1995).

The extent of *A. flavus* invasion in maize grain was assessed using the bright greenish yellow fluorescence (BGYF) test (Busboom and White, 2004; Matumba et al., 2013). After shelling and drying (as described above), 100-kernel random samples were taken for each plot. Kernel samples were arranged in one layer on trays, and the extent of BGYF (FL) was visually assessed in a dark room with 365 nm UV light using the same scale as described above for ear rot score.

**FIGURE 1:**
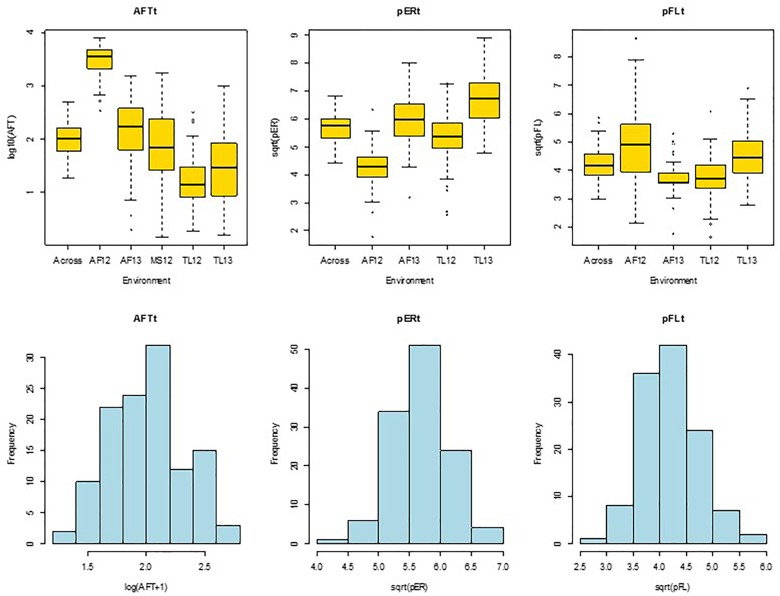
Quartile distribution box plots **(upper)** and histograms **(lower)** of hybrid means for aflatoxin concentration in grain [AFTt, log(AFT+1)], *A. flavus* ear rot symptom scores [pERt, sqrt(pER)], and *A. flavus* colonization of grain measured by bright greenish yellow fluorescence scores [pFLt, sqrt(pFL)]. Across, across environments; AF12, AF13, Agua Fria, 2012 and 2013; TL12, TL13, Tlaltizapan 2012 and 2013; MS12, Mississippi 2012.

Data for ER and FL were obtained for the four Mexican environments only. The ER and FL rating scores were converted to percent ear rot (pER) and percent BGYF (pFL) by equating 0, 25, 50, 75, and 100% to scores of 1, 2, 3, 4, and 5, respectively. The pER and pFL values were transformed using square root to normalize the data distributions prior to statistical analyses (Figure 1). We subsequently refer to the transformed data as pERt and pFLt.

### Quantification of Aflatoxin Concentration in Grain

Aflatoxin concentration in grain was quantified for 50 g sub-samples of ground maize kernels from each plot using VICAM’s AflaTest protocol (Watertown, MA, United States). Briefly, 5 g NaCl were added to a glass blender jar containing the ground grain and 100 ml of 80% methanol was added as a solvent to extract aflatoxin from the grain. This solution was mixed at the high speed of a common kitchen blender for 1 min. The filtrate was then collected from each sample using fluted Whatman #4 filter paper. Ten ml of the filtered extract was added to a clean flask with 40 ml of purified water and this diluted extract was filtered again using a microfiber filter. Column chromatography was performed by passing 2 ml of filtered diluted extract through the AflaTest column. Contaminants were removed by washing the column twice with 5 ml of purified water. One ml of HPLC grade methanol was then added to the chromatography column to elute the aflatoxin. Sub-samples with more than 500 parts per billion (ppb) of aflatoxin were re-tested: (1) for samples with 500–699 ppb, 1 ml of the first filtered extract was added to 49 ml of 15% methanol and then 2 ml of this dilution was passed through a new AflaTest column; (2) for samples with 700–999 ppb, only 1 ml of the dilution was used for the new chromatography; and (3) for samples with >1000 ppb, a 40X dilution was made by diluting 1 ml of the first filtrate in 99 ml of 15% methanol and loading 1 ml of this 40X diluted filtrate into the chromatography column. Aflatoxin concentration (AFT) was expressed in nanograms per gram or ppb, and these data were transformed using log_10_(AFT+1) to normalize the data (Figure 1) prior to statistical analyses. We subsequently refer to the transformed AFT data as AFTt.

### Carotenoid Quantification

Carotenoid concentrations were quantified by Ultra Performance Liquid Chromatography (UPLC) (Muzhingi et al., 2017). Briefly, ethanol was added to 600 mg of finely ground grain samples, followed by saponification and carotenoids extraction using hexane as a solvent. A 30C UPLC column was used for the separation, and quantification of carotenoids used a multi-wavelength detector set at 450 nm. Data collection and processing were conducted using Waters Millennium, 2010 software (Waters Chromatography, Milford, MA, United States). LT, ZX, β-cryptoxanthin (BCX), and all-*trans*-β-carotene (BC) were identified through their characteristic spectra and by comparing their retention times with known standard solutions. Total proVA content (μg g^-1^ of dry matter) was calculated for each sample as the sum of BC plus half of BCX.

### Statistical Analyses

Analyses of variance (ANOVA) were performed for AFTt, pERt and pFLt data for the 120 hybrids at individual and across trial locations. Individual environment ANOVAs used a linear mixed model with replications and incomplete blocks within replications as random effects, and line, tester and line × tester (hybrid) considered as fixed effects. Similarly, for ANOVA across environments, the effects of replicates within environment and incomplete blocks within replicates and environment were considered random effects, whereas lines, testers, and hybrids as well as their interaction with environments were considered fixed effects. These ANOVAs were performed using the MIXED procedure of SAS (SAS 9, 2017).

The hybrids source of variation was sub-divided into variance attributed to testers (T), lines (L) and interaction of L × T. The variance among testers was further sub-divided into variance among hybrids of resistant (R) or susceptible (S) testers, and the contrast between R and S. Similarly, the variance among lines (L) was sub-divided into variance among hybrids of lines with high (Hi) or low (Lo) carotenoids concentrations, and the contrast between Hi and Lo. The contrast R vs. S estimated the significance of differences in AFTt, pERt, and pFLt between hybrids of resistant or susceptible testers, whereas the contrast of Hi vs. Lo estimated the significance of differences in AFTt, pERt, and pFLt between hybrids of lines with high or low concentrations of carotenoids. The contrasts were performed for each trait (AFTt, pERt, and pFLt) for each individual environment and combined across environments using the MIXED procedure together with the CONTRAST command of SAS (SAS 9, 2017).

The classification of the 20 experimental lines as Hi or Lo was based on the average carotenoid concentrations of the F_2_ grain of their six hybrids (Table 1 and Supplementary Table S1). The lines whose hybrids had greater carotenoid value than the average were categorized as Hi, and the others were classified as Lo. This classification was done independently for each carotenoid, BC, BCX, ZX and LT. We used the classification based on carotenoids concentrations in the F2 grain because *A. flavus* inoculations and subsequent AFT, pER, and pFL measurements also used F2 grain.

**Table 1:** Numbers of hybrids and mean carotenoid concentrations (μg g^-1^ dry weight) for F2 grain of hybrids grouped as high (Hi) and low (Lo) for carotenoids concentrations.

	ProVA		BC		BCX		ZX		LT	
					
	*n*	Mean	*n*	Mean	*n*	Mean	*N*	Mean	*n*	Mean
Hi	9	3.59	7	2.04	9	1.39	9	2.82	8	0.73
Lo	11	1.33	13	0.59	11	0.80	11	1.59	12	0.43
Hi/Lo		2.71		3.46		1.74		1.77		1.69
*p* value		<0.0001		<0.0001		<0.0001		<0.0001		<0.0001
Overall mean		2.35		1.10		1.07		2.14		0.55


*ProVA, total provitamin A carotenoids concentration; BC, β-carotene; BCX, β-cryptoxanthin; ZX, zeaxanthin; LT, lutein. N is the number of lines in each group. See Supplementary Table S1 for data for each hybrid.*

Phenotypic and genotypic correlation coefficients among variables were estimated using entry means (for aflatoxin traits) and entry values (for carotenoid traits). Additive main effects and multiplicative interactions (AMMI) (Gauch, 1988) analysis was performed for four traits (BCX, BC, AFTt, pERt) where the hybrid by environments interaction was decomposed into principal components, and a biplot (hybrids and environments) involving the first two principal components was drawn using AGD-R software (Rodríguez et al., 2015).

Repeatability (H*^2^*) across environments was estimated as:

$$H^{2}=\frac{\sigma_{g}^{2}}{\sigma_{g}^{2}+\sigma_{ge}^{2}/l+\sigma_{g}^{2}/rl}$$

Where $\sigma_{g}^{2}$ is the genotypic variance, $\sigma_{ge}^{2}$ is the genotype by environment interaction variance, $\sigma_{g}^{2}$ is error variance, *r* is the number of replications and *l* is the number of environments. The repeatability for individual environment analyses was:

$$H^{2}=\frac{\sigma_{g}^{2}}{\sigma_{g}^{2}+\sigma_{e}^{2}/r}$$

## Results

The data transformations resulted in approximately normal distributions for entry means for AFTt, pERt, and pFLt (Figure 1). Subsequent analyses of variance resulted in moderate to high repeatabilities at individual sites and across locations for AFTt (0.45–0.71 and 0.61), pERt (0.32–0.56 and 0.42) and pFLt (0.23–0.76 and 0.54) (Tables 2–4 and Supplementary Tables S4–S6), indicating that the trials were of good quality. Least squares means for AFTt, pERt and pFLt at individual and across environments are presented in Supplementary Table S7.

**Table 2:** Probabilities of significance (*p*) for *F*-tests for contrasts of hybrids of aflatoxin resistant (R) vs. susceptible (S) testers, and for hybrids with high (Hi) vs. low (Lo) carotenoids concentrations for aflatoxin concentration in grain [AFTt, log(AFT+1)] for analyses of variance at individual and across five trial environments.

Source of variation	Carotenoids	Across (*p*)	AF12 (*p*)	AF13 (*p*)	MS12 (*p*)	TL12 (*p*)	TL13 (*p*)
R vs. S	All	<0.0001	<.0001	0.0011	<0.0001	<0.0001	<0.0001
Hi vs. Lo	ProVA	0.0235	0.2793	0.0002	0.4333	0.0299	0.6399
	BC	<0.0001	0.1620	<0.0001	0.4218	0.0175	0.0503
	BCX	0.0008	0.7214	0.3698	0.0011	0.2961	0.1155
	ZX	<0.0001	0.8773	0.3364	0.0391	0.1530	<0.0001
	LT	<0.0001	0.2564	<0.0001	0.0202	0.0974	<0.0001
*H^2^*		0.61	0.66	0.68	0.71	0.45	0.71
CV (%)		28.15	8.65	26.85	34.39	53.60	45.02


*AF12, AF13, Agua Fria, 2012 and 2013; TL12, TL13, Tlaltizapan 2012 and 2013; MS12, Mississippi 2012. ProVA, total provitamin A; BC, β-carotene; BCX, β-cryptoxanthin; ZX, zeaxanthin; LT, lutein concentration in grain. *H*^2^, repeatability; CV, coefficient of variation.*

**Table 3:** Probabilities of significance (*p*) for *F*-tests for contrasts of hybrids of aflatoxin resistant (R) vs. susceptible (S) testers, and for hybrids with high (Hi) vs. low (Lo) carotenoids concentrations for *A. flavus* ear rot symptom scores [pERt, sqrt(pER)] for analyses of variance at individual and across five trial environments.

Source of variation	Carotenoids	Across (*p*)	AF12 (*p*)	AF13 (*p*)	TL12 (*p*)	TL13 (*p*)
R vs. S	All	<0.0001	0.0001	0.2766	0.0442	0.0008
Hi vs. Lo	ProVA	<0.0001	0.0707	0.0101	0.5812	0.0017
	BC	<0.0001	0.1085	<0.0001	0.6628	0.0477
	BCX	0.0825	0.0004	0.5990	0.7132	0.9981
	ZX	0.4579	0.4143	0.0770	0.6834	0.9740
	LT	<0.0001	0.2999	<0.0001	0.1264	0.1012
H*^2^*		0.42	0.51	0.56	0.32	0.40
CV (%)		21.79	22.75	18.90	24.58	20.39


*AF12, AF13, Agua Fria, 2012 and 2013; TL12, TL13, Tlaltizapan 2012 and 2013; MS12, Mississippi 2012. ProVA, total provitamin A; BC, β-carotene; BCX, β-cryptoxanthin; ZX, zeaxanthin; LT, lutein concentration in grain. *H*^2^, repeatability; CV, coefficient of variation.*

**Table 4:** Probabilities of significance (*p*) for *F*-tests for contrasts of hybrids of aflatoxin resistant (R) vs. susceptible (S) testers, and for hybrids with high (Hi) vs. low (Lo) carotenoids concentrations for *A. flavus* colonization of grain measured by bright greenish yellow fluorescence scores [pFLt, sqrt(pFL)] for analyses of variance at individual and across five trial environments.

Source of variation	Carotenoids	Across (*p*)	AF12 (*p*)	AF13 (*p*)	TL12 (*p*)	TL13 (*p*)
R vs. S	All	<0.0001	<0.0001	0.2285	0.2572	<0.0001
Hi vs. Lo	ProVA	0.2078	0.7548	0.9539	0.9113	0.0762
	BC	0.0142	<0.0001	0.5900	0.4349	0.4506
	BCX	0.1737	0.2260	0.7437	0.4114	0.8838
	ZX	0.0155	0.8212	0.1718	0.0587	0.0537
	LT	0.0054	0.6818	0.0054	0.0624	0.1601
*H^2^*		0.54	0.76	0.44	0.23	0.49
CV (%)		24.89	23.41	17.18	32.09	25.22


*AF12, AF13, Agua Fria, 2012 and 2013; TL12, TL13, Tlaltizapan 2012 and 2013; MS12, Mississippi 2012. ProVA, total provitamin A; BC, β-carotene; BCX, β-cryptoxanthin; ZX, zeaxanthin; LT, lutein concentration in grain. *H*^2^, repeatability; CV, coefficient of variation.*

The hybrids differed significantly (*p* < 0.001) for AFTt, pERt, and pFLt in all single- and across-location analyses. Environment (E) effects were also highly significant (*p* < 0.001) for all traits (Supplementary Tables S4A, S5A, S6A), with the most notable difference that mean AFTt was greater at AF12 than other sites (Figure 1). The highly significant effect of hybrids confirms that the 120 hybrids differed for their resistance or susceptibility to *A. flavus*. Further, the significant (*p* < 0.001) variance for all traits due to testers, lines, and lines × testers effects (Supplementary Tables S4A, S5A, S6A), indicates the significance of additive or general combining ability (GCA), and non-additive or specific combining ability (SCA) effects on *A. flavus* resistance of the hybrids. The highly significant (*p* < 0.001) contrasts of resistant versus susceptible testers (R vs. S) for AFTt, pERt, and pFLt in the across-environment analyses (Tables 2–4) confirm that the hybrids of resistant testers were indeed significantly more resistant to *A. flavus* than the hybrids of susceptible testers. Significant (*p* < 0.001) interactions of environments with hybrid, tester, line, and line × tester effects indicate that GCA and SCA effects varied between environments.

Analyses of variance for carotenoids concentrations identified significant (*p* < 0.0001 or *p* < 0.001) environmental effects and significant (*p* < 0.0001) differences among hybrids for all traits (proVA, BC, BCX, ZX, and LT) (ANOVA not shown). More interestingly, the contrasts of hybrids with high versus low carotenoid grain concentration (Hi vs. Lo) for total proVA (*p* < 0.05), BC, BCX, ZX, and LT (*p* < 0.001) were significant for across-environment analyses for AFTt (Table 2), indicating that carotenoid concentrations affected aflatoxin accumulation in grain. Similarly, the Hi vs. Lo contrasts for proVA, BC, and LT were significant for pERt (*p* < 0.001) (Table 3), and the Hi vs. Lo contrasts for BC, ZX (*p* < 0.05) and LT (*p* < 0.01) were significant for pFLt (Table 4).

Analysis of the 60 hybrids of susceptible testers indicated that hybrids with larger concentrations (Hi) of proVA, BC and BCX always had significantly less ear rot and aflatoxin than hybrids with smaller (Lo) concentrations of these carotenoids (Table 5). This relationship was generally also significant for hybrids of *A. flavus* resistant tester lines. By contrast, hybrids with larger concentrations of ZX and LT were generally more susceptible to *A. flavus* infection than those with smaller concentrations of these carotenoids.

**Table 5:** Contrasts of aflatoxin concentration (AFTt), ear rot (pERt), and fluorescence scores (pFLt) between hybrids with high (Hi) vs. low (Lo) grain concentrations of five carotenoids, analyzed for all 120 hybrids and independently for sub-sets of 60 hybrids of *Aspergillus flavus* resistant (R) or 60 hybrids of susceptible (S) tester lines.

Trait	Carotenoid	All hybrids		R testers		S testers	
				
		Hi	Lo	Hi	Lo	Hi	Lo
AFTt	ProVA	1.99*	2.05	1.80	1.85	2.18*	2.26
	BC	1.94***	2.07	1.74***	1.87	2.13***	2.27
	BCX	1.98***	2.06	1.79	1.86	2.16***	2.27
	ZX	2.08	1.97***	1.89	1.78**	2.28	2.17**
	LT	2.12	1.96***	1.89	1.78**	2.35	2.13***
							
pERt	ProVA	5.42***	5.65	5.28**	5.51	5.57**	5.79
	BC	5.39***	5.63	5.26**	5.48	5.52**	5.79
	BCX	5.49	5.59	5.40	5.41	5.60*	5.77
	ZX	5.57	5.53	5.45	5.36	5.68	5.70
	LT	5.71	5.44***	5.56	5.30**	5.85	5.59**
							
pFLt	ProVA	4.25	4.19	4.12	3.99*	4.38	4.40
	BC	4.14*	4.26	4.02	4.06	4.25**	4.47
	BCX	4.18	4.25	4.04	4.06	4.32	4.45
	ZX	4.28	4.17*	4.10	4.01	4.47	4.33*
	LT	4.30	4.16**	4.11	4.01	4.49	4.32*


*^∗^, ^∗∗^, ^∗∗∗^ indicate the mean which is significantly (*p* < 0.05, <0.01, or <0.001, respectively) superior for the resistance trait (AFTt, pERt, or pFLt) in the respective pair-wise contrast, e.g., Hi vs. Lo proVA concentration among all hybrids, among hybrids of *A. flavus* resistant testers, or among hybrids of *A. flavus* susceptible testers. Pairs of means without indication of significance were not significantly different (*p* < 0.05).*

Some of the contrasts of R vs. S testers and Hi vs. Lo lines from single environment analysis were not significant, indicating the greater power of the combined analyses. The interaction effects of E × (R vs. S testers) were significant for AFTt and pFLt (*p* < 0.001), indicating that the magnitude of differences among the means of hybrids of R and S testers varied between environments (Table 6). Significant interactions occurred for E × (Hi vs. Lo proVA, BC, ZX, and LT lines) for AFTt, E × (Hi vs. Lo BC and LT lines) for pERt, and E × (Hi vs. Lo BC lines) for pFLt.

**Table 6:** Probabilities of significance (*p*) for *F*-tests for interaction contrasts of environment (E) by hybrids of aflatoxin resistant (R) vs. susceptible (S) testers, and for E by hybrids with high (Hi) vs. low (Lo) carotenoids concentrations for aflatoxin concentration in grain [AFTt, log(AFT + 1)], *A. flavus* ear rot symptom scores [pERt, sqrt(pER)], and *A. flavus* colonization of grain measured by bright greenish yellow fluorescence scores [pFLt, sqrt(pFL)] for analyses of variance across five (AFTt) or four (pERt and pFLt) trial environments.

Source of variation	Carotenoids (*p*)	AFTt (*p*)	pERt (*p*)	pFLt (*p*)
E × (R vs. S)	All	<0.0001	0.3201	<0.0001
E × (Hi vs. Lo)	ProVA	0.0016	0.1485	0.3845
	BC	0.0352	0.0369	0.0006
	BCX	0.0733	0.2110	0.7936
	ZX	0.0157	0.4490	0.2649
	LT	<0.0001	0.0306	0.6668


*ProVA, total provitamin A; BC, β-carotene; BCX, β-cryptoxanthin; ZX, zeaxanthin; LT, lutein concentration in grain.*

Estimates of genotypic correlation coefficients indicate that AFTt and pERt were negatively associated with concentrations of BCX (*p* < 0.01), BC (*p* < 0.05), and proVA (*p* < 0.05 and *p* < 0.01, respectively) in grain (Table 7). Phenotypic correlation coefficients for carotenoids concentrations with *A. flavus* infection traits were only significant (*p* < 0.05) for BCX with AFTt and pERt. The phenotypic and genotypic correlation coefficients among carotenoid concentrations, and among *A. flavus* infection parameters were generally as expected. The phenotypic correlation between the two visually-scored traits, pERt and pFLt, was moderate (*r*_P_ = 0.56, *p* < 0.001), but the genotypic correlation coefficient between these traits was high (*r*_G_ = 0.93, *p* < 0.001). Moreover, the correlation coefficients between the visually-scored traits and the quantitative estimate of aflatoxin concentration (AFTt) were all very strong (*p* < 0.001) and positive (*r*_P_ = 0.56–0.69; *r*_G_ = 0.89–1.00).

**Table 7:** Pearson phenotypic correlation (above diagonal) and genotypic correlation (below diagonal) coefficients and among carotenoid and aflatoxin traits for F2 grain of 120 hybrids grown at four environments.

	LT	ZX	BCX	BC	ProVA	pERt	pFLt	AFTt
LT		0.99***	0.68***	0.30**	0.40***	-0.06 ns	0.07 ns	-0.10 ns
ZX	0.99***		0.72***	0.35***	0.46***	-0.10 ns	0.05 ns	-0.11 ns
BCX	0.68***	0.73***		0.50***	0.65***	-0.19*	0.01 ns	-0.18*
BC	0.29**	0.35***	0.50***		0.98***	-0.12 ns	0.05 ns	-0.13 ns
ProVA	0.39***	0.45***	0.64***	0.98***		-0.15 ns	0.05 ns	-0.16 ns
pERt	-0.09 ns	-0.14 ns	-0.30***	-0.23*	-0.26**		0.56***	0.56***
pFLt	0.11 ns	0.09 ns	0.02 ns	0.08 ns	0.08 ns	0.93***		0.69***
AFTt	-0.16 ns	-0.17 ns	-0.28**	-0.18*	-0.23*	0.89***	1.00***	


*ProVA, total provitamin A; BC, β-carotene; BCX, β-cryptoxanthin; ZX, zeaxanthin; LT, lutein concentration in grain. ns, not significant, *p* < 0.05; ^∗^, ^∗∗^, and ^∗∗∗^ = significant *p* < 0.05, 0.01, and 0.001, respectively.*

**FIGURE 2:**
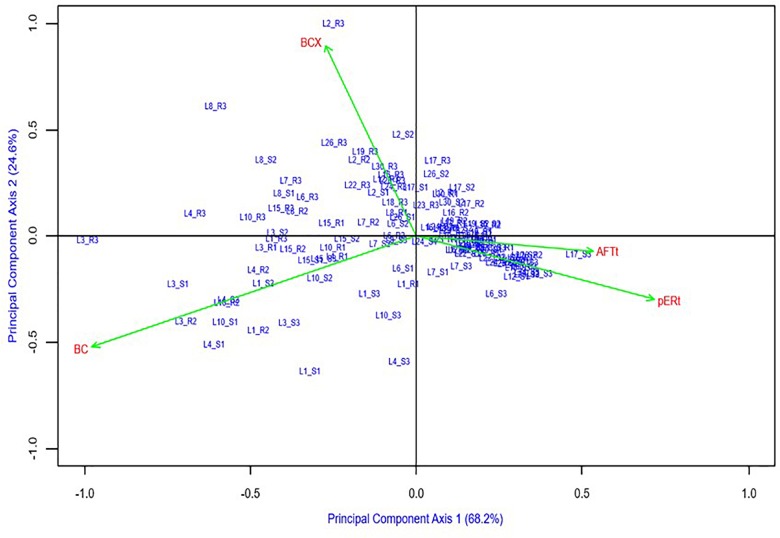
Additive main effects and multiplicative interactions (AMMI) biplot of hybrids and four traits: aflatoxin concentration in grain [AFTt, log(AFT+1)], *A. flavus* ear rot symptom scores [pERt, sqrt(pER)], β-carotene (BC), β-cryptoxanthin (BCX) concentration in grain.

The AMMI biplot for main effects and interactions of genotypes and traits visibly separated aflatoxin (AFTt and pERt) from carotenoid concentrations (BCX and BC) along PCA1, which explained 68% of the variation (Figure 2). PCA2, which explained an additional 25% of the variation, indicated a factor that associated AFTt and pERt positively with BC and negatively with BCX.

## Discussion

Our finding of large variation for AFTt, pERt, and pFLt among environments, and even among micro-environments (replications), is consistent with previous reports of large environmental influence on *A. flavus* infection during pre-harvest, and on aflatoxin accumulation during both pre- and post-harvest (Arunyanark et al., 2010; Mayfield et al., 2011; Warburton et al., 2011). This highlights the importance of using multiple replications and environments to achieve repeatable results when studying *A. flavus* resistance-related traits. The normal frequency distributions for the transformed data (Figure 1), and the moderate to high repeatability estimates from analyses of variance (Tables 2–4), indicate the reliability of our results. Secondly, the clear separation of the hybrids’ grain as resistant or susceptible to *A. flavus* (R vs. S contrast, Tables 2–4) justified the further analyses of associated effects of grain carotenoids concentrations with *A. flavus* resistance.

Although the trial environments were diverse and used different *A. flavus* inocula, we treated them as fixed in ANOVA analyses because there were only five of them. However, the five environments included lowland tropical, mid-altitude tropical, and temperate ecologies, and their respective fungal isolate diversity. We speculated that the scope of inference for our findings is broader than our five trial environments, and found that ANOVA using environments as random effects produced same results as the model treating environments as fixed, i.e., all sources of variation were significant at the same broad probability levels (NS, *p* < 0.05, *p* < 0.01, *p* < 0.001, etc.) (Supplementary Table S8). We conclude that our findings can be extended, with caution until validated more widely, beyond the five experimental environments.

Maize grain with higher concentrations of proVA, BC, and BCX had less aflatoxin contamination (AFTt) than grain with smaller carotenoid concentrations (Table 5). This superior aflatoxin resistance of hybrids with high versus low concentrations of proVA, BC, and BCX was statistically significant among the 60 hybrids formed with *A. flavus* susceptible tester lines. For the 60 hybrids formed with *A. flavus* resistant parent tester lines, however, only hybrids with contrasting BC concentrations differed significantly for AFTt in grain. The fact that only BC provided a statistically significant additional aflatoxin resistance benefit to hybrids formed with *A. flavus* resistant parents may have been because the differences in high versus low carotenoids concentrations were much larger for BC than for BCX and total proVA (Table 1). These results indicate that increased concentrations of BC, BCX, and proVA carotenoids can be a valuable first line of defense against aflatoxin contamination of grain. This further suggests that proVA biofortified maize, or any maize with increased concentrations of these carotenoids can offer an important health benefit for consumers affected by both VAD and chronic exposure to aflatoxin contaminated maize products. This is particularly important for sub-Saharan Africa, where a large health burden of exposure to aflatoxin (see discussion above, or e.g., Williams et al., 2004; Wild, 2007), prevalence of VAD (Muthayya et al., 2013), and large dependence on maize as a staple food converge.

The fact that hybrids with smaller concentrations of ZX and LT were significantly more resistant to aflatoxin than hybrids with larger concentrations of these carotenoids is likely due to their competing roles with proVA, BC and BCX within the carotenoid biosynthetic pathway. Provitamin A biofortification breeding programs have selected for alleles of (1) the *LcyE* gene (Harjes et al., 2008) that decrease flux toward the alpha-branch of the carotenoid biosynthetic pathway, and hence decrease LT concentration in grain, and (2) the *CrtRB1* gene (Babu et al., 2013) that reduce flux from BC toward BCX and ZX (Pixley et al., 2013).

Maize grain with higher concentrations of BC had smaller mean pERt and pFLt than grain with low BC concentration (Tables 3, 4). However, although generally favorable, the effects of carotenoids concentrations on the means for the visually-assessed indicators of aflatoxin contamination (pERt and pFLt), were weaker and less consistent than for AFTt. This result is consistent with lower repeatability for these visually-assessed traits compared to AFTt.

The GCA effects of lines and testers, as well as their interactions with environments were highly significant for AFTt, pERt, and pFLt (*p* < 0.001) (Supplementary Table S4A, S5A, S6A). The line × tester (SCA) effects were highly significant for AFTt and pFLt (*p* < 0.001), but not for pERt (*p* = 0.056). This indicates the importance of both additive and non-additive gene actions for the inheritance of aflatoxin resistance in maize. These results are consistent with experience that breeding for aflatoxin resistance is complex, requiring selection for specific hybrid combinations that avail dominance and epistatic gene actions. The fact that breeding directly for aflatoxin resistance is very challenging adds importance to our findings that increasing carotenoids concentrations had desirable effect for reducing aflatoxin concentrations in grain. Breeding for increased carotenoids concentrations in maize grain is relatively straightforward (Pixley et al., 2013; Tanumihardjo et al., 2017).

The significant genotypic correlation coefficients for proVA, BC, and BCX with AFTt and pERt (Table 7) indicate that these relationships are rooted in common genetic factors. The magnitude of these genotypic correlations was small [*r*_G_ = 0.18 (*p* < 0.05) to 0.30 (*p* < 0.01)], and the potential to achieve double health benefits from proVA biofortified maize therefore requires further validation.

The AMMI biplot helps visualize the strong negative genetic correlations between aflatoxin traits (AFTt and pERt) and carotenoids concentrations (BC and BCX) on the X-axis (68% of variance), and a weaker factor negatively associating BCX and positively associating BC concentration with aflatoxin traits (Y-axis, 25% of variance) (Figure 2). Large cumulative percentage for the two PC axes (93%) indicates that the biplot captured most of the genotype-by-trait variation. The combined effects of the two PCA axes suggest that while BC had strongest influence (PCA1), BCX contributed an important additional mechanism of action against aflatoxin production or accumulation (PCA2). This finding adds a new dimension - aflatoxin resistance - to support nutritional arguments presented by Dhliwayo et al. (2014) and Taleon et al. (2017) for reconsidering current proVA biofortification breeding strategies that strongly reduce BCX in favor of accumulating more BC (Babu et al., 2013; Zunjare et al., 2018). We propose to further investigate these relationships using maize lines with wider ranges of BCX and BC concentrations than used herein (Table 1).

At the time of this study, CIMMYT had no orange maize lines with characterized resistances to *Aspergillus* ear rot and aflatoxin accumulation for possible use as testers. Otherwise, using orange maize lines as testers would have produced larger levels of carotenoid concentrations in grains and might have improved the investigation of their effects on *Aspergillus* ear rot and aflatoxin accumulation. However, if orange testers had been used, differences for carotenoid concentrations might have been confounded with differences in resistance genes among the testers, complicating the interpretation of results.

Several secondary traits associated with aflatoxin accumulation have been proposed for use in indirect selection, e.g., rating for insect damage, ear injury, husk cover (Betrán et al., 2002), fungus biomass estimation by qPCR (Mideros et al., 2009), and drought tolerance (Arunyanark et al., 2010). Campbell and White (1995) suggested using visual selection for low *Aspergillus* ear rot symptom scores (pERt in our study) to select lines with greater aflatoxin resistance, thereby avoiding more expensive aflatoxin assays. Visual ratings of *Aspergillus* ear rot symptoms and of BGYF substance (pFLt in our study) are simpler and much less expensive than aflatoxin quantification, which requires expensive chemical reagents, specific equipment and technical skills.

Although the genotypic correlation of *Aspergillus* ear rot symptom score (pERt) with AFTt was highly significant (*r* = 0.89, *p* < 0.001) for the 120 hybrids studied herein, experience and literature (e.g., Walker and White, 2001; Henry et al., 2009) suggest that this trait is not a very reliable indicator of aflatoxin concentration in grain. The strong positive genotypic correlation for AFTt with pFLt (*r* = 1.00, *p* < 0.001) (Table 7), suggests that pFLt may be useful for rapid indirect assessment of potential aflatoxin accumulation. Successful use of the BGYF test requires technical skills for sampling (Campbell et al., 1986) and visual scoring (Dickens and Whitaker, 1981; Henry et al., 2009), but its simplicity, low cost and greater reliability than visual ear rot symptom scores make it an appealing candidate for use as a secondary trait and predictor of aflatoxin concentration.

In conclusion, this is the first published report documenting significant reduction in aflatoxin contamination for maize with conventionally-bred levels of carotenoids. This result was found using maize hybrids with carotenoid concentrations that are one-half, one-third, or only one-fourth as large as more recent hybrids developed by CIMMYT’s proVA biofortification breeding program (Pixley et al., 2013; Andersson et al., 2017; Sowa et al., 2017). The relative ease of breeding for increased carotenoid concentrations as compared to breeding for aflatoxin resistance in maize make this finding especially significant as part of a solution to aflatoxin contamination problems for maize. Furthermore, because the antioxidant effects of carotenoids on reducing aflatoxin production are non-enzymatic, it is likely that these act also in grain during post-harvest, when *A. flavus* infection and aflatoxin production are a serious concern and when most breeding strategies, including transgenic HIGS strategies are expected to be ineffective (Gressel and Polturak, 2018).

Future research should assess (1) whether stronger effects on reducing *A. flavus* infection and aflatoxin contamination have accrued by breeding maize with further-increased concentrations of BC and BCX and (2) whether significant aflatoxin-reducing effects also occur during post-harvest exposure of grain to *A. flavus*. Also, and although aflatoxin is of greatest global concern, it will be valuable to assess whether increased concentrations of BC and BCX confer advantages for reducing infection and production of mycotoxins by *Fusarium* spp.

The results herein demonstrate that BC, BCX, and proVA concentrations already present in biofortified hybrids can provide an advantage for reducing aflatoxin levels in maize. Thus, maize with increased content of proVA carotenoids may offer double health benefits by reducing aflatoxin concentrations while contributing to reduce vitamin A deficiency in affected maize consuming populations.

## Data Availability Statement

Datasets are available on request: The raw data supporting the conclusions of this manuscript will be made available by the authors, without undue reservation, to any qualified researcher.

## Author Contributions

WS, PH, and KP contributed equally as first authors. KP, NP-R, and PH contributed conception and design of the study, developed the hybrids, and oversaw the trials in Mexico. GW implemented the trial at MS and enabled the VICAM aflatoxin analyses for those samples. NP-R, PH, and WS organized the data. PH conducted the VICAM aflatoxin analyses for all Mexican sites and performed preliminary data analyses and interpretation as part of her Ph.D. thesis under guidance of KP. NP-R oversaw the carotenoids analyses. JC and WS performed the statistical analyses. All authors contributed to manuscript revision, read and approved the submitted version.

## Conflict of Interest Statement

The authors declare that the research was conducted in the absence of any commercial or financial relationships that could be construed as a potential conflict of interest.

## Abbreviations

AFT: aflatoxin concentration in grain
BC: β-carotene
BCX: β-cryptoxanthin
LT: lutein
pER: *A. flavus* ear rot symptom scores
pFL: *A. flavus* colonization of grain measured by bright greenish yellow fluorescence scores
ProVA: total provitamin A carotenoids concentration
ZX: zeaxanthin
