# Supplementary material for: Provitamin A Carotenoids in Grain Reduce Aflatoxin Contamination of Maize While Combating Vitamin A Deficiency
Source: Front Plant Sci. 2019 Jan 29;10:30. doi: 10.3389/fpls.2019.00030 (PMC6369730; doi:10.3389/fpls.2019.00030)
Supplement: Supplementary file 1 [file Table_1.pdf]

# Provitamin A carotenoids in grain reduce aflatoxin contamination of maize while combating vitamin A deficiency

**Willy B. Suwarno[1,2], Pattama Hannok[1,3†], Natalia Palacios-Rojas[1], Gary Windham[4], José Crossa[1], Kevin V. Pixley[1,3*†]**

Supplementary Table S1. Pedigree of the lines and carotenoids mean of the lines (1 location) and their respective hybrids (4 locations)

| Line ID | Pedigree | ProVA | | BC | | BCX | | ZX | | LT |
|---|---|---|---|---|---|---|---|---|---|---|
| | | Line | Hybrid | Line | Hybrid | Line | Hybrid | Line | Hybrid | Hybrid |
| L1 | [MAS[MSR/312]-117-2-2-1-B-B-B/[BETASYN]BC1-4-1-4-#//MAS[MSR/312]-117-2-2-1-B-B-B/[BETASYN]BC1-4-1-4/KUISYN#]-7-2-1-B | 16.94 | 4.05 H | 13.61 | 2.26 H | 6.65 | 0.99 L | 18.58 | 4.01 H | 0.98 H |
| L2 | (Florida A plus Syn-FS2-2-1-B-B/(KU1409/DE3/KU1409)S2-18-2-B)-B-3(MAS:L4H1)-2-B-B-B | 18.79 | 2.75 H | 14.13 | 1.04 L | 9.31 | 2.05 H | 14.17 | 4.20 H | 1.04 H |
| L3 | (KUI carotenoid syn-FS11-1-1-B-B-B/(KU1409/DE3/KU1409)S2-18-2-B)-B-3(MAS:L4H1)-1-B-B-B | 40.05 | 5.04 H | 35.20 | 2.82 H | 9.69 | 1.46 H | 1.14 | 2.23 H | 0.52 L |
| L4 | (KUI carotenoid syn-FS17-3-2-B-B-B/(KU1409/DE3/KU1409)S2-18-2-B)-B-1(MAS:L4H1)-1-B-B-B | 39.52 | 4.87 H | 34.94 | 2.69 H | 9.17 | 1.38 H | 2.72 | 2.87 H | 0.68 H |
| L5 | SAM4(ProA)BC1/KUISyn#-1-39-1-3-B-B-B | 15.07 | 2.44 H | 11.64 | 1.13 H | 6.85 | 1.06 L | 3.08 | 1.12 L | 0.30 L |
| L6 | [CML445/[BETASYN]BC1-2-3-3-1//CML445/[BETASYN]BC1-2-3-3/KUI+SC55SYN#]-9-1-1-2-B | 14.83 | 2.41 H | 10.31 | 1.09 L | 9.03 | 1.03 L | 17.07 | 2.13 L | 0.53 L |
| L7 | (P591c4 1y2 GEN F12-1-1-1-B-B-B/KUI carotenoid syn-FS25-3-2-B)-9-3-1-B | 14.12 | 3.19 H | 8.55 | 1.44 H | 11.14 | 1.64 H | 16.65 | 2.21 H | 0.55 L |
| L8 | (KUI carotenoid syn-FS17-3-2-B-B-B/(KU1409/DE3/KU1409)S2-18-2-B)-B-1(MAS:L4H1)-5-B | 35.74 | 4.39 H | 30.96 | 2.35 H | 9.55 | 1.25 H | 1.59 | 2.13 L | 0.52 L |
| L9 | ((DTPYC9-F65-2-3-1-1-B-BxDTPYC9-F65-2-2-1-1-B-B)xDTPYC9-F46-1-7-1-1-B-B-B)-B-B-3-2-B-B | 11.61 | 1.39 L | 7.85 | 0.58 L | 7.52 | 0.73 L | 17.31 | 1.36 L | 0.33 L |
| L10 | SAM4(ProA)BC1/KUISyn#-1-39-1-1-B-B | 15.02 | 3.18 H | 11.93 | 1.58 H | 6.18 | 1.13 H | 2.61 | 1.36 L | 0.35 L |
| L11 | ([[[K64R/G16SR]-39-1/[K64R/G16SR]-20-2]-5-1-2-B*4/CML390]-B-38-1-B-7-#/[BETASYN]BC1-1-1-1-#/Carotenoid Syn3-FS11-4-3-B)-7-3-1-B-B | 3.04 | 1.35 L | 1.32 | 0.5 L | 3.45 | 0.9 L | 12.66 | 2.69 H | 0.70 H |
| L12 | ([[[NAW5867/P30SR]-40-1/[NAW5867/P30SR]-114-2]-16-2-2-B-2-B/CML395-6]-B-20-1-B-3-#/[BETASYN]BC1-3-1-1-#/CML297)-9-1-1-B-B | 2.61 | 1.47 L | 0.32 | 0.49 L | 4.58 | 1.2 H | 10.81 | 2.23 H | 0.60 H |
| L13 | ([GQL5/[GQL5/CML202]F2-1sx]-3-1-2-B/[BETASYN]BC1-6-1-1/KUISYN#-B)-B-1-2-B-B | 1.37 | 1.19 L | 0.39 | 0.45 L | 1.97 | 0.71 L | 9.43 | 1.87 L | 0.54 L |
| L14 | [CML445/[BETASYN]BC1-6-3-1-#//CML445/[BETASYN]BC1-6-3-1/KUISYN#]-8-2-1-5-B | 2.84 | 1.34 L | 1.01 | 0.49 L | 3.68 | 0.94 L | 15.84 | 2.05 L | 0.56 H |
| L15 | [MAS[MSR/312]-117-2-2-1-B-B-B/[BETASYN]BC1-11-1-4-#//MAS[MSR/312]-117-2-2-1-B-B-B/[BETASYN]BC1-11-1-4/FloridaASYN#]-6-1-2-B | 1.55 | 1.37 L | 0.41 | 0.55 L | 2.28 | 0.81 L | 8.23 | 2.23 H | 0.59 H |
| L16 | CML451 | 0.79 | 0.94 L | 0.00 | 0.38 L | 1.57 | 0.52 L | 2.66 | 1.47 L | 0.38 L |
| L17 | OBATANPA-SRc1F3(balbulk1)-#bal/[BETASYN]BC1-67-1-2-B-B-B-B-B-B-B-B | 0.70 | 0.89 L | 0.00 | 0.32 L | 1.40 | 0.54 L | 0.55 | 0.75 L | 0.29 L |
| L18 | [[GQL5/[GQL5/[MSRXPOOL9]C1F2-205-1(OSU23i)-5-3-X-X-1-B-B]F2-4sx]-8-6-B-B//[BETASYN]BC1-14-2-1-#/[GQL5/[GQL5/[MSRXPOOL9]C1F2-205-1(OSU23i)-5-3-X-X-1-B-B]F2-4sx]-8-6-B-B/[BETASYN]BC1-14-2-1/KUISYN#]-9-3-3-2-B | 1.88 | 1.01 L | 0.64 | 0.41 L | 2.47 | 0.56 L | 3.55 | 1.35 L | 0.36 L |
| L19 | (CML300/CML486)-7-2-3-B-B | 6.78 | 1.97 L | 2.57 | 0.76 L | 8.43 | 1.27 H | 10.84 | 1.93 L | 0.50 L |
| L20 | ([[[K64R/G16SR]-39-1/[K64R/G16SR]-20-2]-5-1-2-B)*4/CML390]-B-38-1-B-7-#/[BETASYN]BC1-1-1-1-#/KUI carotenoid syn-FS25-3-2-B)-4-3-2-B-B | 5.65 | 1.67 L | 2.77 | 0.61 L | 5.76 | 1.16 H | 8.50 | 2.67 H | 0.67 H |

Supplementary Table S2. Pedigree of the testers and aflatoxin (AFT) mean of their respective hybrids

| Tester ID | Pedigree | Hybrid AFT (ng $g^{-1}$) | Characteristic |
|---|---|---|---|
| R1 | CML495 | 81 | R |
| R2 | CML442 | 89 | R |
| R3 | CML321 | 66 | R |
| S1 | DTPWC9-F31-1-3-1-1-B-B-B | 139 | S |
| S2 | CML340 | 167 | S |
| S3 | Pob.SEW-HG"B"c0F39-1-2-2-1-B-B | 218 | S |

*R = aflatoxin resistant; S = aflatoxin susceptible*

Supplementary Table S3. Planting, inoculation and harvest months, and average daily weather conditions

| Environment | Planting date | Inoculation date | Harvesting date | High temp (°C) | Low temp (°C) | Rainfall (mm) | Relative humidity (%) |
|---|---|---|---|---|---|---|---|
| AF 2012 | April | June | August | 34 | 23 | 6.3 | 84 |
| AF 2013 | January | April | June | 31 | 20 | 3.4 | 81 |
| TL 2012 | June | September | November | 32 | 17 | 3.1 | 64 |
| TL 2013 | February | April | June | 34 | 15 | 1.5 | 51 |
| MS 2012 | May | July | September | 32 | 23 | 4.6 | NA |

AF = Agua Fria; TL = Tlaltizapan; MS = Mississippi

Supplementary Table S4A. Summary of complete anova **across environments** for AFTt

| Source of | | | ProVA | | BC | | BCX | | ZX | | LT | |
|---|---|---|---|---|---|---|---|---|---|---|---|---|
| variation | DF | ProbF | DF | ProbF | DF | ProbF | DF | ProbF | DF | ProbF | DF | ProbF |
| Env | 4 | <.0001 | | | | | | | | | | |
| Rep(Env) | 14 | 0.0004 | | | | | | | | | | |
| Block(Env × Rep) | 209 | <.0001 | | | | | | | | | | |
| Hybrids | 119 | <.0001 | | | | | | | | | | |
| Testers | 5 | <.0001 | | | | | | | | | | |
| Tester R | 2 | <.0001 | | | | | | | | | | |
| Tester S | 2 | 0.1322 | | | | | | | | | | |
| R vs S | 1 | <.0001 | | | | | | | | | | |
| Lines | 19 | <.0001 | | | | | | | | | | |
| Line H | | | 8 | <.0001 | 6 | 0.0004 | 8 | <.0001 | 8 | <.0001 | 7 | 0.0014 |
| Line L | | | 10 | <.0001 | 12 | <.0001 | 10 | <.0001 | 10 | <.0001 | 11 | <.0001 |
| H vs L | | | 1 | 0.0235 | 1 | <.0001 | 1 | 0.0008 | 1 | <.0001 | 1 | <.0001 |
| Line × Tester | 95 | <.0001 | | | | | | | | | | |
| Env × Hybrids | 476 | <.0001 | | | | | | | | | | |
| Env × Testers | 20 | <.0001 | | | | | | | | | | |
| Env × Tester R | 8 | <.0001 | | | | | | | | | | |
| Env × Tester S | 8 | <.0001 | | | | | | | | | | |
| Env × (R vs S) | 1 | <.0001 | | | | | | | | | | |
| Env × Lines | 76 | <.0001 | | | | | | | | | | |
| Env × Line H | | | 32 | 0.0115 | 24 | 0.0301 | 32 | <.0001 | 32 | 0.0003 | 28 | 0.0456 |
| Env × Line L | | | 40 | <.0001 | 48 | <.0001 | 40 | 0.0001 | 40 | 0.0006 | 44 | <.0001 |
| Env × (H vs L) | | | 1 | 0.0016 | 1 | 0.0352 | 1 | 0.0733 | 1 | 0.0157 | 1 | <.0001 |
| Env × Line × Tester | 380 | <.0001 | | | | | | | | | | |
| Residual | 1425 | | | | | | | | | | | |

Supplementary Table S4B. Summary of complete ANOVA by environment for AFTt

| Env | Source of | | | ProVA | | BC | | BCX | | ZX | | LT | |
|---|---|---|---|---|---|---|---|---|---|---|---|---|---|
| | variation | DF | ProbF | DF | ProbF | DF | ProbF | DF | ProbF | DF | ProbF | DF | ProbF |
| AF12 | Rep | 3 | 0.0023 | | | | | | | | | | |
| | Block(Rep) | 44 | 0.2784 | | | | | | | | | | |
| | Hybrids | 119 | <.0001 | | | | | | | | | | |
| | Testers | 5 | <.0001 | | | | | | | | | | |
| | Tester R | 2 | 0.0002 | | | | | | | | | | |
| | Tester S | 2 | 0.0909 | | | | | | | | | | |
| | R vs S | 1 | <.0001 | | | | | | | | | | |
| | Lines | 19 | <.0001 | | | | | | | | | | |
| | Line H | | | 8 | <.0001 | 6 | 0.0002 | 8 | <.0001 | 8 | 0.0201 | 7 | 0.0086 |
| | Line L | | | 10 | 0.0001 | 12 | <.0001 | 10 | <.0001 | 10 | <.0001 | 11 | <.0001 |
| | H vs L | | | 1 | 0.2793 | 1 | 0.162 | 1 | 0.7214 | 1 | 0.8773 | 1 | 0.2564 |
| | Line × Tester | 95 | 0.0029 | | | | | | | | | | |
| | Residual | 313 | | | | | | | | | | | |
| AF13 | Rep | 3 | 0.0777 | | | | | | | | | | |
| | Block(Rep) | 44 | <.0001 | | | | | | | | | | |
| | Hybrids | 119 | <.0001 | | | | | | | | | | |
| | Testers | 5 | <.0001 | | | | | | | | | | |
| | Tester R | 2 | 0.4207 | | | | | | | | | | |
| | Tester S | 2 | <.0001 | | | | | | | | | | |
| | R vs S | 1 | 0.0011 | | | | | | | | | | |
| | Lines | 19 | <.0001 | | | | | | | | | | |
| | Line H | | | 8 | 0.0003 | 6 | 0.0011 | 8 | 0.0012 | 8 | 0.0003 | 7 | 0.6546 |
| | Line L | | | 10 | 0.0260 | 12 | 0.0161 | 10 | <.0001 | 10 | 0.0009 | 11 | <.0001 |
| | H vs L | | | 1 | 0.0002 | 1 | <.0001 | 1 | 0.3698 | 1 | 0.3364 | 1 | <.0001 |
| | Line × Tester | 95 | 0.0003 | | | | | | | | | | |
| | Residual | 307 | . | | | | | | | | | | |
| MS12 | Rep | 2 | 0.0046 | | | | | | | | | | |
| | Block(Rep) | 33 | 0.6242 | | | | | | | | | | |
| | Hybrids | 119 | <.0001 | | | | | | | | | | |
| | Testers | 5 | <.0001 | | | | | | | | | | |
| | Tester R | 2 | 0.0003 | | | | | | | | | | |
| | Tester S | 2 | 0.0207 | | | | | | | | | | |
| | R vs S | 1 | <.0001 | | | | | | | | | | |
| | Lines | 19 | <.0001 | | | | | | | | | | |
| | Line H | | | 8 | 0.0842 | 6 | 0.3356 | 8 | 0.0143 | 8 | 0.0001 | 7 | 0.0019 |
| | Line L | | | 10 | 0.0023 | 12 | 0.0004 | 10 | 0.0085 | 10 | 0.0972 | 11 | 0.032 |
| | H vs L | | | 1 | 0.4333 | 1 | 0.4218 | 1 | 0.0011 | 1 | 0.0391 | 1 | 0.0202 |
| | Line × Tester | 95 | 0.0041 | | | | | | | | | | |
| | Residual | 184 | | | | | | | | | | | |

| Env | Source of | | | ProVA | | BC | | BCX | | ZX | | LT | |
|---|---|---|---|---|---|---|---|---|---|---|---|---|---|
| | variation | | | DF | ProbF | DF | ProbF | DF | ProbF | DF | ProbF | DF | ProbF |
| TL12 | Rep | 3 | 0.4259 | | | | | | | | | | |
| | Block(Rep) | 44 | 0.0409 | | | | | | | | | | |
| | Hybrids | 119 | 0.0001 | | | | | | | | | | |
| | Testers | 5 | <.0001 | | | | | | | | | | |
| | Tester R | 2 | 0.5534 | | | | | | | | | | |
| | Tester S | 2 | 0.0017 | | | | | | | | | | |
| | R vs S | 1 | <.0001 | | | | | | | | | | |
| | Lines | 19 | 0.0864 | | | | | | | | | | |
| | Line H | | | 8 | 0.6080 | 6 | 0.7217 | 8 | 0.4435 | 8 | 0.4016 | 7 | 0.2094 |
| | Line L | | | 10 | 0.0206 | 12 | 0.0856 | 10 | 0.1086 | 10 | 0.3665 | 11 | 0.3937 |
| | H vs L | | | 1 | 0.0299 | 1 | 0.0175 | 1 | 0.2961 | 1 | 0.1530 | 1 | 0.0974 |
| | Line × Tester | 95 | 0.0373 | | | | | | | | | | |
| | Residual | 313 | | | | | | | | | | | |
| TL13 | Rep | 3 | 0.0227 | | | | | | | | | | |
| | Block(Rep) | 44 | 0.0504 | | | | | | | | | | |
| | Hybrids | 119 | <.0001 | | | | | | | | | | |
| | Testers | 5 | <.0001 | | | | | | | | | | |
| | Tester R | 2 | <.0001 | | | | | | | | | | |
| | Tester S | 2 | 0.9366 | | | | | | | | | | |
| | R vs S | 1 | <.0001 | | | | | | | | | | |
| | Lines | 19 | <.0001 | | | | | | | | | | |
| | Line H | | | 8 | 0.0014 | 6 | 0.0171 | 8 | <.0001 | 8 | 0.0040 | 7 | 0.3650 |
| | Line L | | | 10 | <.0001 | 12 | <.0001 | 10 | 0.0012 | 10 | 0.0014 | 11 | 0.0004 |
| | H vs L | | | 1 | 0.6399 | 1 | 0.0503 | 1 | 0.1155 | 1 | <.0001 | 1 | <.0001 |
| | Line × Tester | 95 | 0.0014 | | | | | | | | | | |
| | Residual | 308 | | | | | | | | | | | |

Supplementary Table S5A. Summary of complete ANOVA across environments for pERt

| Source of | | | ProVA | | BC | | BCX | | ZX | | LT | |
|---|---|---|---|---|---|---|---|---|---|---|---|---|
| variation | | | DF | ProbF | DF | ProbF | DF | ProbF | DF | ProbF | DF | ProbF |
| Env | 3 | <.0001 | | | | | | | | | | |
| Rep(Env) | 12 | 0.0001 | | | | | | | | | | |
| Block(Env × Rep) | 176 | <.0001 | | | | | | | | | | |
| Hybrids | 119 | <.0001 | | | | | | | | | | |
| Testers | 5 | <.0001 | | | | | | | | | | |
| Tester R | 2 | 0.0001 | | | | | | | | | | |
| Tester S | 2 | 0.7072 | | | | | | | | | | |
| R vs S | 1 | <.0001 | | | | | | | | | | |
| Lines | 19 | <.0001 | | | | | | | | | | |
| Line H | | | 8 | <.0001 | 6 | <.0001 | 8 | <.0001 | 8 | <.0001 | 7 | 0.5356 |
| Line L | | | 10 | 0.0564 | 12 | 0.0226 | 10 | 0.0583 | 10 | <.0004 | 11 | <.0001 |
| H vs L | | | 1 | <.0001 | 1 | <.0001 | 1 | 0.0825 | 1 | 0.4579 | 1 | <.0001 |
| Line × Tester | 95 | 0.0559 | | | | | | | | | | |
| Env × Hybrids | 357 | <.0001 | | | | | | | | | | |
| Env × Testers | 15 | 0.0002 | | | | | | | | | | |
| Env × Tester R | 6 | 0.0004 | | | | | | | | | | |
| Env × Tester S | 6 | 0.1717 | | | | | | | | | | |
| Env × (R vs S) | 1 | 0.3201 | | | | | | | | | | |
| Env × Lines | 57 | <.0001 | | | | | | | | | | |
| Env × Line H | | | 24 | 0.0376 | 18 | 0.2569 | 24 | 0.0020 | 24 | 0.0002 | 21 | 0.0006 |
| Env × Line L | | | 30 | <.0001 | 36 | <.0001 | 30 | 0.0013 | 30 | 0.0052 | 33 | 0.0010 |
| Env × (H vs L) | | | 1 | 0.1485 | 1 | 0.0369 | 1 | 0.2110 | 1 | 0.4490 | 1 | 0.0306 |
| Env × Line × Tester | 285 | 0.0048 | | | | | | | | | | |
| Residual | 1241 | | | | | | | | | | | |

Supplementary Table S5B. Summary of complete ANOVA by environment for pERt

| Env | Source of variation | | | ProVA | | BC | | BCX | | ZX | | LT | |
|---|---|---|---|---|---|---|---|---|---|---|---|---|---|
| | | | | DF | ProbF | DF | ProbF | DF | ProbF | DF | ProbF | DF | ProbF |
| AF12 | Rep | 3 | 0.0121 | | | | | | | | | | |
| | Block(Rep) | 44 | 0.6770 | | | | | | | | | | |
| | Hybrids | 119 | <.0001 | | | | | | | | | | |
| | Testers | 5 | 0.0010 | | | | | | | | | | |
| | Tester R | 2 | 0.0474 | | | | | | | | | | |
| | Tester S | 2 | 0.3726 | | | | | | | | | | |
| | R vs S | 1 | <.0001 | | | | | | | | | | |
| | Lines | 19 | 0.0192 | | | | | | | | | | |
| | Line H | | | 8 | 0.0131 | 6 | 0.0195 | 8 | 0.2731 | 8 | 0.1712 | 7 | 0.7135 |
| | Line L | | | 10 | 0.7633 | 12 | 0.7361 | 10 | 0.6900 | 10 | 0.3563 | 11 | 0.0234 |
| | H vs L | | | 1 | 0.0707 | 1 | 0.1085 | 1 | 0.0004 | 1 | 0.4143 | 1 | 0.2999 |
| | Line × Tester | 95 | <.0001 | | | | | | | | | | |
| | Residual | 313 | | | | | | | | | | | |
| AF13 | Rep | 3 | 0.0126 | | | | | | | | | | |
| | Block(Rep) | 44 | 0.0001 | | | | | | | | | | |
| | Hybrids | 119 | <.0001 | | | | | | | | | | |
| | Testers | 5 | <.0001 | | | | | | | | | | |
| | Tester R | 2 | 0.0004 | | | | | | | | | | |
| | Tester S | 2 | 0.1235 | | | | | | | | | | |
| | R vs S | 1 | 0.2766 | | | | | | | | | | |
| | Lines | 19 | <.0001 | | | | | | | | | | |
| | Line H | | | 8 | 0.0003 | 6 | 0.0076 | 8 | <.0001 | 8 | <.0001 | 7 | 0.0007 |
| | Line L | | | 10 | 0.0014 | 12 | <.0001 | 10 | 0.0019 | 10 | 0.0104 | 11 | 0.0018 |
| | H vs L | | | 1 | 0.0101 | 1 | <.0001 | 1 | 0.5990 | 1 | 0.0770 | 1 | <.0001 |
| | Line × Tester | 95 | 0.0171 | | | | | | | | | | |
| | Residual | 307 | | | | | | | | | | | |
| TL12 | Rep | 3 | 0.0001 | | | | | | | | | | |
| | Block(Rep) | 44 | 0.0116 | | | | | | | | | | |
| | Hybrids | 119 | 0.0064 | | | | | | | | | | |
| | Testers | 5 | 0.0830 | | | | | | | | | | |
| | Tester R | 2 | 0.3563 | | | | | | | | | | |
| | Tester S | 2 | 0.2228 | | | | | | | | | | |
| | R vs S | 1 | 0.0442 | | | | | | | | | | |
| | Lines | 19 | 0.0016 | | | | | | | | | | |
| | Line H | | | 8 | 0.0342 | 6 | 0.0228 | 8 | 0.0038 | 8 | 0.0030 | 7 | 0.0267 |
| | Line L | | | 10 | 0.0215 | 12 | 0.0503 | 10 | 0.2385 | 10 | 0.1736 | 11 | 0.0821 |
| | H vs L | | | 1 | 0.5812 | 1 | 0.6628 | 1 | 0.7132 | 1 | 0.6834 | 1 | 0.1264 |
| | Line × Tester | 95 | 0.1108 | | | | | | | | | | |
| | Residual | 313 | | | | | | | | | | | |

| Env | Source of variation | | | ProVA | | BC | | BCX | | ZX | | LT | |
|---|---|---|---|---|---|---|---|---|---|---|---|---|---|
| | | | | DF | ProbF | DF | ProbF | DF | ProbF | DF | ProbF | DF | ProbF |
| TL13 | Rep | 3 | <.0001 | | | | | | | | | | |
| | Block(Rep) | 44 | 0.1127 | | | | | | | | | | |
| | Hybrids | 119 | 0.0006 | | | | | | | | | | |
| | Testers | 5 | <.0001 | | | | | | | | | | |
| | Tester R | 2 | <.0001 | | | | | | | | | | |
| | Tester S | 2 | 0.7508 | | | | | | | | | | |
| | R vs S | 1 | 0.0008 | | | | | | | | | | |
| | Lines | 19 | <.0001 | | | | | | | | | | |
| | Line H | | | 8 | 0.3025 | 6 | 0.4083 | 8 | 0.0018 | 8 | 0.0548 | 7 | 0.3936 |
| | Line L | | | 10 | 0.0082 | 12 | 0.0019 | 10 | 0.0135 | 10 | 0.0010 | 11 | <.0001 |
| | H vs L | | | 1 | 0.0017 | 1 | 0.0477 | 1 | 0.9981 | 1 | 0.9740 | 1 | 0.1012 |
| | Line × Tester | 95 | 0.6731 | | | | | | | | | | |
| | Residual | 308 | | | | | | | | | | | |

Supplementary Table S6A. Summary of complete ANOVA across environments for pFLt

| Source of | | | ProVA | | BC | | BCX | | ZX | | LT | |
|---|---|---|---|---|---|---|---|---|---|---|---|---|
| variation | | | DF | ProbF | DF | ProbF | DF | ProbF | DF | ProbF | DF | ProbF |
| Env | 3 | 0.0004 | | | | | | | | | | |
| Rep(Env) | 12 | <.0001 | | | | | | | | | | |
| Block(Env × Rep) | 176 | 0.0055 | | | | | | | | | | |
| Hybrids | 119 | <.0001 | | | | | | | | | | |
| Testers | 5 | <.0001 | | | | | | | | | | |
| Tester R | 2 | <.0001 | | | | | | | | | | |
| Tester S | 2 | 0.0727 | | | | | | | | | | |
| R vs S | 1 | <.0001 | | | | | | | | | | |
| Lines | 19 | <.0001 | | | | | | | | | | |
| Line H | | | 8 | <.0001 | 6 | 0.0247 | 8 | <.0001 | 8 | <.0001 | 7 | <.0001 |
| Line L | | | 10 | <.0001 | 12 | <.0001 | 10 | <.0001 | 10 | <.0001 | 11 | <.0001 |
| H vs L | | | 1 | 0.2078 | 1 | 0.0142 | 1 | 0.1737 | 1 | 0.0155 | 1 | 0.0054 |
| Line × Tester | 95 | 0.0002 | | | | | | | | | | |
| Env × Hybrids | 357 | <.0001 | | | | | | | | | | |
| Env × Testers | 15 | <.0001 | | | | | | | | | | |
| Env × Tester R | 6 | 0.0239 | | | | | | | | | | |
| Env × Tester S | 6 | 0.8138 | | | | | | | | | | |
| Env × (R vs S) | 1 | <.0001 | | | | | | | | | | |
| Env × Lines | 57 | <.0001 | | | | | | | | | | |
| Env × Line H | | | 24 | <.0001 | 18 | 0.0033 | 24 | 0.0012 | 24 | 0.1724 | 21 | 0.2642 |
| Env × Line L | | | 30 | 0.0002 | 36 | <.0001 | 30 | <.0001 | 30 | <.0001 | 33 | <.0001 |
| Env × (H vs L) | | | 1 | 0.3845 | 1 | 0.0006 | 1 | 0.7936 | 1 | 0.2649 | 1 | 0.6668 |
| Env × Line × Tester | 285 | 0.0019 | | | | | | | | | | |
| Residual | 1241 | | | | | | | | | | | |

Supplementary Table S6B. Summary of complete ANOVA by environment for pFLt

| Env | Source of variation | | | ProVA | | BC | | BCX | | ZX | | LT | |
|---|---|---|---|---|---|---|---|---|---|---|---|---|---|
| | | | | DF | ProbF | DF | ProbF | DF | ProbF | DF | ProbF | DF | ProbF |
| AF12 | Rep | 3 | 0.6056 | | | | | | | | | | |
| | Block(Rep) | 44 | 0.3867 | | | | | | | | | | |
| | Hybrids | 119 | <.0001 | | | | | | | | | | |
| | Testers | 5 | <.0001 | | | | | | | | | | |
| | Tester R | 2 | <.0001 | | | | | | | | | | |
| | Tester S | 2 | 0.7943 | | | | | | | | | | |
| | R vs S | 1 | <.0001 | | | | | | | | | | |
| | Lines | 19 | <.0001 | | | | | | | | | | |
| | Line H | | | 8 | <.0001 | 6 | 0.0003 | 8 | <.0001 | 8 | 0.0004 | 7 | 0.0014 |
| | Line L | | | 10 | <.0001 | 12 | <.0001 | 10 | <.0001 | 10 | <.0001 | 11 | <.0001 |
| | H vs L | | | 1 | 0.7548 | 1 | <.0001 | 1 | 0.2260 | 1 | 0.8212 | 1 | 0.6818 |
| | Line × Tester | 95 | <.0001 | | | | | | | | | | |
| | Residual | 313 | | | | | | | | | | | |
| AF13 | Rep | 3 | 0.5058 | | | | | | | | | | |
| | Block(Rep) | 44 | 0.5373 | | | | | | | | | | |
| | Hybrids | 119 | <.0001 | | | | | | | | | | |
| | Testers | 5 | <.0001 | | | | | | | | | | |
| | Tester R | 2 | 0.0301 | | | | | | | | | | |
| | Tester S | 2 | 0.0008 | | | | | | | | | | |
| | R vs S | 1 | 0.2285 | | | | | | | | | | |
| | Lines | 19 | 0.0004 | | | | | | | | | | |
| | Line H | | | 8 | 0.1018 | 6 | 0.1273 | 8 | 0.0617 | 8 | 0.0080 | 7 | 0.0946 |
| | Line L | | | 10 | 0.0153 | 12 | 0.0133 | 10 | 0.0274 | 10 | 0.0619 | 11 | 0.1050 |
| | H vs L | | | 1 | 0.9539 | 1 | 0.5900 | 1 | 0.7437 | 1 | 0.1718 | 1 | 0.0054 |
| | Line × Tester | 95 | 0.0222 | | | | | | | | | | |
| | Residual | 307 | | | | | | | | | | | |
| TL12 | Rep | 3 | <.0001 | | | | | | | | | | |
| | Block(Rep) | 44 | 0.0518 | | | | | | | | | | |
| | Hybrids | 119 | 0.0428 | | | | | | | | | | |
| | Testers | 5 | 0.0493 | | | | | | | | | | |
| | Tester R | 2 | 0.0350 | | | | | | | | | | |
| | Tester S | 2 | 0.5628 | | | | | | | | | | |
| | R vs S | 1 | 0.2572 | | | | | | | | | | |
| | Lines | 19 | 0.1810 | | | | | | | | | | |
| | Line H | | | 8 | 0.9080 | 6 | 0.9301 | 8 | 0.8049 | 8 | 0.2424 | 7 | 0.0894 |
| | Line L | | | 10 | 0.0075 | 12 | 0.0092 | 10 | 0.0220 | 10 | 0.2416 | 11 | 0.1979 |
| | H vs L | | | 1 | 0.9113 | 1 | 0.4349 | 1 | 0.4114 | 1 | 0.0587 | 1 | 0.0624 |
| | Line × Tester | 95 | 0.1291 | | | | | | | | | | |
| | Residual | 313 | . | | | | | | | | | | |

| Env | Source of | | | ProVA | | BC | | BCX | | ZX | | LT | |
|---|---|---|---|---|---|---|---|---|---|---|---|---|---|
| | variation | | | DF | ProbF | DF | ProbF | DF | ProbF | DF | ProbF | DF | ProbF |
| TL13 | Rep | 3 | 0.0007 | | | | | | | | | | |
| | Block(Rep) | 44 | 0.0115 | | | | | | | | | | |
| | Hybrids | 119 | <.0001 | | | | | | | | | | |
| | Testers | 5 | <.0001 | | | | | | | | | | |
| | Tester R | 2 | 0.0002 | | | | | | | | | | |
| | Tester S | 2 | 0.7243 | | | | | | | | | | |
| | R vs S | 1 | <.0001 | | | | | | | | | | |
| | Lines | 19 | <.0001 | | | | | | | | | | |
| | Line H | | | 8 | 0.1183 | 6 | 0.4634 | 8 | 0.1802 | 8 | 0.0018 | 7 | 0.0063 |
| | Line L | | | 10 | <.0001 | 12 | <.0001 | 10 | <.0001 | 10 | <.0001 | 11 | <.0001 |
| | H vs L | | | 1 | 0.0762 | 1 | 0.4506 | 1 | 0.8838 | 1 | 0.0537 | 1 | 0.1601 |
| | Line × Tester | 95 | 0.5669 | | | | | | | | | | |
| | Residual | 308 | | | | | | | | | | | |

Supplementary Table S7A. Least square means at 5 environments and across-environments for aflatoxin contamination of grain (AFTt) for 120 hybrids formed by crossing 6 inbred tester lines (R1-R3 are *A. flavus* resistant, and S1-S3 are susceptible) with 20 inbred maize lines with varying concentrations of carotenoids in grain (L1-L20).

| Hybrid | T x L | Across | AF12 | AF13 | MS12 | TL12 | TL13 |
|---|---|---|---|---|---|---|---|
| 1 | R1xL1 | 1.99 | 3.43 | 2.41 | 2.14 | 1.25 | 0.72 |
| 2 | R2xL1 | 1.64 | 3.67 | 1.51 | 1.49 | 0.61 | 0.91 |
| 3 | S1xL1 | 2.54 | 3.63 | 1.69 | 2.64 | 2.35 | 2.41 |
| 4 | S2xL1 | 2.54 | 3.55 | 2.72 | 2.96 | 1.28 | 2.16 |
| 5 | S3xL1 | 2.09 | 3.56 | 2.38 | 2.15 | 0.33 | 2.03 |
| 6 | R3xL1 | 1.98 | 3.71 | 2.56 | 0.18 | 1.59 | 1.86 |
| 7 | R1xL2 | 2.15 | 3.78 | 2.73 | 2.08 | 1.15 | 1.03 |
| 8 | R2xL2 | 1.95 | 3.58 | 1.58 | 1.60 | 1.59 | 1.43 |
| 9 | S1xL2 | 2.16 | 3.68 | 2.03 | 2.24 | 1.01 | 1.84 |
| 10 | S2xL2 | 2.65 | 3.70 | 2.83 | 2.29 | 1.85 | 2.59 |
| 11 | S3xL2 | 2.23 | 3.63 | 2.83 | 1.83 | 1.05 | 1.79 |
| 12 | R3xL2 | 2.15 | 3.69 | 2.33 | 1.43 | 1.50 | 1.82 |
| 13 | R1xL3 | 1.80 | 3.00 | 1.54 | 2.60 | 0.84 | 1.03 |
| 14 | R2xL3 | 1.80 | 3.59 | 1.41 | 1.68 | 1.28 | 1.04 |
| 15 | S1xL3 | 1.71 | 3.45 | 1.38 | 1.80 | 1.61 | 0.32 |
| 16 | S2xL3 | 1.93 | 3.33 | 2.30 | 1.39 | 0.72 | 1.90 |
| 17 | S3xL3 | 2.04 | 3.66 | 2.23 | 1.85 | 1.35 | 1.12 |
| 18 | R3xL3 | 1.55 | 3.60 | 1.99 | 0.75 | 1.14 | 0.26 |
| 19 | R1xL4 | 1.93 | 3.06 | 2.20 | 1.84 | 1.02 | 1.52 |
| 20 | R2xL4 | 2.01 | 3.41 | 2.19 | 1.91 | 1.07 | 1.46 |
| 21 | S1xL4 | 2.00 | 3.68 | 1.47 | 1.89 | 0.93 | 2.04 |
| 22 | S2xL4 | 2.26 | 3.34 | 2.93 | 1.72 | 1.13 | 2.18 |
| 23 | S3xL4 | 2.61 | 3.68 | 2.92 | 2.68 | 1.97 | 1.80 |
| 24 | R3xL4 | 1.52 | 3.31 | 2.32 | 0.65 | 0.64 | 0.69 |
| 25 | R1xL5 | 1.80 | 3.31 | 1.68 | 2.17 | 0.70 | 1.14 |
| 26 | R2xL5 | 1.75 | 3.64 | 1.89 | 1.93 | 0.38 | 0.91 |
| 27 | S1xL5 | 2.14 | 3.70 | 0.92 | 2.87 | 1.51 | 1.73 |
| 28 | S2xL5 | 2.04 | 3.62 | 1.80 | 1.62 | 1.49 | 1.67 |

| | | | | | | | |
|---|---|---|---|---|---|---|---|
| 29 | S3xL5 | 2.61 | 3.36 | 2.80 | 2.53 | 1.89 | 2.45 |
| 30 | R3xL5 | 1.22 | 3.15 | 0.80 | 0.48 | 0.89 | 0.79 |
| 31 | R1xL6 | 2.21 | 3.63 | 1.87 | 2.62 | 1.19 | 1.74 |
| 32 | R2xL6 | 1.63 | 3.46 | 1.31 | 1.64 | 0.50 | 1.23 |
| 33 | S1xL6 | 2.37 | 3.80 | 1.69 | 2.86 | 1.54 | 1.97 |
| 34 | S2xL6 | 2.14 | 3.59 | 2.35 | 2.80 | 0.45 | 1.54 |
| 35 | S3xL6 | 2.53 | 3.79 | 2.83 | 2.42 | 1.96 | 1.64 |
| 36 | R3xL6 | 1.82 | 3.58 | 2.16 | 1.62 | 0.75 | 1.00 |
| 37 | R1xL7 | 2.03 | 3.25 | 1.68 | 2.44 | 0.80 | 1.99 |
| 38 | R2xL7 | 1.77 | 3.88 | 1.99 | 1.92 | 0.32 | 0.74 |
| 39 | S1xL7 | 2.00 | 3.27 | 0.52 | 2.88 | 1.41 | 1.94 |
| 40 | S2xL7 | 1.93 | 3.47 | 1.87 | 2.33 | 1.10 | 0.86 |
| 41 | S3xL7 | 2.30 | 3.51 | 2.56 | 2.17 | 1.57 | 1.67 |
| 42 | R3xL7 | 1.52 | 3.35 | 1.27 | 1.39 | 1.01 | 0.56 |
| 43 | R1xL8 | 1.84 | 3.51 | 2.25 | 1.87 | 1.03 | 0.53 |
| 44 | R2xL8 | 1.83 | 3.67 | 1.50 | 1.35 | 0.59 | 2.07 |
| 45 | S1xL8 | 1.95 | 3.77 | 1.84 | 1.59 | 0.99 | 1.55 |
| 46 | S2xL8 | 2.20 | 3.79 | 3.12 | 1.69 | 0.75 | 1.63 |
| 47 | S3xL8 | 2.53 | 3.82 | 2.89 | 2.19 | 1.77 | 1.95 |
| 48 | R3xL8 | 1.69 | 3.58 | 2.52 | 0.69 | 1.00 | 0.68 |
| 49 | R1xL9 | 2.33 | 3.60 | 2.50 | 2.05 | 1.75 | 1.77 |
| 50 | R2xL9 | 2.24 | 3.50 | 2.12 | 1.73 | 1.45 | 2.40 |
| 51 | S1xL9 | 2.49 | 3.57 | 1.66 | 3.11 | 1.88 | 2.21 |
| 52 | S2xL9 | 2.40 | 3.63 | 2.70 | 2.35 | 1.20 | 2.10 |
| 53 | S3xL9 | 2.72 | 3.80 | 2.73 | 2.42 | 1.64 | 3.03 |
| 54 | R3xL9 | 1.84 | 3.60 | 2.56 | 1.01 | 1.10 | 0.93 |
| 55 | R1xL10 | 1.51 | 3.24 | 2.06 | 0.80 | 0.55 | 0.92 |
| 56 | R2xL10 | 1.55 | 2.86 | 1.43 | 2.02 | 0.71 | 0.75 |
| 57 | S1xL10 | 2.00 | 3.48 | 0.96 | 2.94 | 0.79 | 1.85 |
| 58 | S2xL10 | 2.04 | 3.08 | 2.79 | 1.61 | 1.30 | 1.43 |
| 59 | S3xL10 | 1.41 | 3.30 | 1.54 | 0.73 | 1.06 | 0.43 |
| 60 | R3xL10 | 1.86 | 2.97 | 2.50 | 1.83 | 1.06 | 0.93 |

| | | | | | | | |
|---|---|---|---|---|---|---|---|
| 61 | R1xL11 | 1.62 | 2.71 | 1.72 | 1.91 | 0.60 | 1.16 |
| 62 | R2xL11 | 2.13 | 3.58 | 2.57 | 2.14 | 0.76 | 1.60 |
| 63 | S1xL11 | 2.38 | 3.52 | 2.20 | 2.07 | 2.03 | 2.08 |
| 64 | S2xL11 | 2.34 | 3.21 | 2.65 | 2.60 | 1.37 | 1.88 |
| 65 | S3xL11 | 2.23 | 3.64 | 2.35 | 2.31 | 1.38 | 1.49 |
| 66 | R3xL11 | 1.56 | 2.81 | 1.98 | 1.43 | 0.76 | 0.82 |
| 67 | R1xL12 | 1.72 | 3.00 | 2.46 | 0.89 | 0.95 | 1.31 |
| 68 | R2xL12 | 1.76 | 3.67 | 1.63 | 0.37 | 1.18 | 1.93 |
| 69 | S1xL12 | 2.14 | 3.54 | 1.97 | 2.02 | 1.05 | 2.12 |
| 70 | S2xL12 | 2.55 | 3.77 | 3.03 | 2.47 | 1.33 | 2.15 |
| 71 | S3xL12 | 2.51 | 3.74 | 3.01 | 1.33 | 2.33 | 2.12 |
| 72 | R3xL12 | 1.88 | 3.44 | 2.93 | 0.81 | 1.08 | 1.16 |
| 73 | R1xL13 | 1.71 | 3.86 | 2.03 | 1.00 | 1.16 | 0.52 |
| 74 | R2xL13 | 2.11 | 3.79 | 2.32 | 1.51 | 1.40 | 1.52 |
| 75 | S1xL13 | 2.16 | 3.34 | 2.47 | 2.20 | 0.82 | 1.96 |
| 76 | S2xL13 | 1.91 | 3.68 | 2.16 | 1.20 | 1.01 | 1.50 |
| 77 | S3xL13 | 2.24 | 3.90 | 2.82 | 1.58 | 1.16 | 1.75 |
| 78 | R3xL13 | 1.39 | 3.09 | 1.80 | 1.07 | 0.46 | 0.53 |
| 79 | R1xL14 | 1.75 | 2.51 | 2.17 | 1.84 | 1.09 | 1.16 |
| 80 | R2xL14 | 1.75 | 3.33 | 1.78 | 1.61 | 0.86 | 1.19 |
| 81 | S1xL14 | 2.49 | 3.68 | 2.33 | 3.27 | 1.24 | 1.93 |
| 82 | S2xL14 | 2.09 | 3.29 | 1.96 | 2.40 | 1.59 | 1.19 |
| 83 | S3xL14 | 2.23 | 3.74 | 2.66 | 2.61 | 1.20 | 0.94 |
| 84 | R3xL14 | 1.42 | 3.18 | 2.28 | 0.70 | 0.53 | 0.40 |
| 85 | R1xL15 | 2.33 | 3.77 | 2.25 | 2.56 | 1.59 | 1.51 |
| 86 | R2xL15 | 2.34 | 3.56 | 2.50 | 2.34 | 1.31 | 1.98 |
| 87 | S1xL15 | 2.50 | 3.55 | 1.89 | 2.70 | 2.02 | 2.33 |
| 88 | S2xL15 | 2.54 | 3.51 | 2.51 | 2.52 | 1.53 | 2.62 |
| 89 | S3xL15 | 2.66 | 3.48 | 2.86 | 2.62 | 2.50 | 1.83 |
| 90 | R3xL15 | 2.04 | 3.71 | 1.93 | 2.19 | 1.00 | 1.35 |
| 91 | R1xL16 | 1.60 | 3.26 | 2.48 | 0.27 | 1.13 | 0.85 |
| 92 | R2xL16 | 1.87 | 3.79 | 2.59 | 1.08 | 1.03 | 0.85 |

| | | | | | | | |
|---|---|---|---|---|---|---|---|
| 93 | S1xL16 | 2.03 | 3.28 | 2.56 | 1.98 | 1.17 | 1.14 |
| 94 | S2xL16 | 2.01 | 3.52 | 2.74 | 1.26 | 1.36 | 1.19 |
| 95 | S3xL16 | 2.16 | 3.65 | 2.23 | 1.97 | 1.37 | 1.58 |
| 96 | R3xL16 | 1.98 | 3.00 | 2.72 | 2.62 | 0.92 | 0.64 |
| 97 | R1xL17 | 1.75 | 3.14 | 1.97 | 1.68 | 1.10 | 0.86 |
| 98 | R2xL17 | 1.79 | 3.53 | 1.82 | 1.84 | 0.82 | 0.95 |
| 99 | S1xL17 | 2.62 | 3.40 | 2.22 | 3.18 | 1.92 | 2.37 |
| 100 | S2xL17 | 1.83 | 3.08 | 2.32 | 1.64 | 1.17 | 0.95 |
| 101 | S3xL17 | 2.04 | 3.43 | 1.72 | 1.56 | 1.57 | 1.89 |
| 102 | R3xL17 | 1.72 | 3.26 | 0.06 | 2.88 | 1.25 | 1.13 |
| 103 | R1xL18 | 2.02 | 3.40 | 2.72 | 1.50 | 0.99 | 1.49 |
| 104 | R2xL18 | 2.00 | 3.74 | 2.23 | 1.52 | 1.40 | 1.11 |
| 105 | S1xL18 | 1.98 | 3.72 | 1.65 | 1.73 | 1.56 | 1.25 |
| 106 | S2xL18 | 1.75 | 3.52 | 2.89 | 0.63 | 0.78 | 0.93 |
| 107 | S3xL18 | 2.22 | 3.74 | 2.01 | 2.15 | 1.27 | 1.91 |
| 108 | R3xL18 | 1.70 | 3.31 | 2.98 | 1.13 | 0.91 | 0.19 |
| 109 | R1xL19 | 1.69 | 3.63 | 2.09 | 0.92 | 1.28 | 0.54 |
| 110 | R2xL19 | 1.69 | 3.43 | 1.93 | 1.24 | 1.11 | 0.73 |
| 111 | S1xL19 | 2.26 | 3.57 | 2.02 | 2.55 | 1.75 | 1.41 |
| 112 | S2xL19 | 2.16 | 3.75 | 2.62 | 1.90 | 0.82 | 1.70 |
| 113 | S3xL19 | 2.07 | 3.84 | 2.10 | 1.49 | 1.79 | 1.16 |
| 114 | R3xL19 | 1.46 | 3.78 | 1.47 | 0.54 | 1.28 | 0.21 |
| 115 | R1xL20 | 1.81 | 3.18 | 1.78 | 1.77 | 0.91 | 1.41 |
| 116 | R2xL20 | 2.09 | 3.68 | 2.22 | 2.00 | 1.20 | 1.35 |
| 117 | S1xL20 | 2.22 | 3.36 | 2.01 | 2.61 | 1.54 | 1.55 |
| 118 | S2xL20 | 2.18 | 3.40 | 2.24 | 2.23 | 1.05 | 1.98 |
| 119 | S3xL20 | 2.44 | 3.84 | 2.60 | 2.55 | 1.06 | 2.16 |
| 120 | R3xL20 | 1.79 | 3.27 | 3.04 | 1.20 | 0.87 | 0.53 |

Supplementary Table S7B. Least square means at 4 environments and across-environments for ear rot symptom score (pERt) for 120 hybrids formed by crossing 6 inbred tester lines (R1-R3 are *A. flavus* resistant, and S1-S3 are susceptible) with 20 inbred maize lines with varying concentrations of carotenoids in grain (L1-L20).

| Hybrid | T x L | Across | AF12 | AF13 | TL12 | TL13 |
|---|---|---|---|---|---|---|
| 1 | R1xL1 | 6.03 | 5.57 | 6.50 | 5.54 | 6.53 |
| 2 | R2xL1 | 5.58 | 3.89 | 6.60 | 5.53 | 6.28 |
| 3 | S1xL1 | 6.25 | 4.57 | 5.56 | 6.08 | 8.80 |
| 4 | S2xL1 | 5.19 | 3.90 | 5.22 | 5.35 | 6.28 |
| 5 | S3xL1 | 5.23 | 5.47 | 6.10 | 2.61 | 6.73 |
| 6 | R3xL1 | 5.16 | 3.79 | 5.71 | 4.86 | 6.27 |
| 7 | R1xL2 | 5.51 | 2.60 | 7.69 | 6.47 | 5.29 |
| 8 | R2xL2 | 5.55 | 3.92 | 5.93 | 4.86 | 7.50 |
| 9 | S1xL2 | 5.45 | 3.46 | 6.60 | 5.27 | 6.47 |
| 10 | S2xL2 | 5.57 | 4.32 | 6.29 | 4.60 | 7.08 |
| 11 | S3xL2 | 5.91 | 4.67 | 7.00 | 5.39 | 6.59 |
| 12 | R3xL2 | 5.63 | 4.28 | 6.34 | 5.10 | 6.82 |
| 13 | R1xL3 | 4.68 | 2.66 | 4.56 | 5.43 | 6.06 |
| 14 | R2xL3 | 5.70 | 5.01 | 5.27 | 5.18 | 7.35 |
| 15 | S1xL3 | 4.98 | 4.22 | 4.51 | 5.93 | 5.28 |
| 16 | S2xL3 | 5.39 | 3.46 | 5.16 | 5.74 | 7.20 |
| 17 | S3xL3 | 5.18 | 4.18 | 5.02 | 5.85 | 5.66 |
| 18 | R3xL3 | 5.15 | 4.52 | 5.01 | 5.66 | 5.42 |
| 19 | R1xL4 | 5.08 | 2.74 | 5.42 | 5.22 | 6.95 |
| 20 | R2xL4 | 5.70 | 4.96 | 5.21 | 5.11 | 7.51 |
| 21 | S1xL4 | 5.75 | 4.59 | 5.53 | 6.41 | 6.47 |
| 22 | S2xL4 | 5.92 | 3.92 | 6.32 | 6.17 | 7.26 |
| 23 | S3xL4 | 6.44 | 4.94 | 7.01 | 7.22 | 6.58 |
| 24 | R3xL4 | 5.24 | 4.22 | 5.30 | 5.18 | 6.24 |
| 25 | R1xL5 | 5.71 | 3.94 | 6.81 | 4.86 | 7.22 |
| 26 | R2xL5 | 5.73 | 4.19 | 6.07 | 4.82 | 7.85 |
| 27 | S1xL5 | 6.09 | 3.90 | 6.68 | 6.62 | 7.16 |
| 28 | S2xL5 | 5.90 | 3.97 | 6.36 | 6.28 | 7.00 |

| | | | | | | |
|---|---|---|---|---|---|---|
| 29 | S3xL5 | 6.18 | 4.59 | 6.05 | 7.10 | 6.97 |
| 30 | R3xL5 | 5.10 | 3.80 | 4.63 | 5.41 | 6.54 |
| 31 | R1xL6 | 5.54 | 4.44 | 5.73 | 6.29 | 5.71 |
| 32 | R2xL6 | 5.24 | 3.84 | 6.64 | 5.06 | 5.41 |
| 33 | S1xL6 | 6.08 | 4.99 | 6.61 | 5.36 | 7.34 |
| 34 | S2xL6 | 5.71 | 4.20 | 6.76 | 5.43 | 6.43 |
| 35 | S3xL6 | 5.73 | 4.59 | 6.38 | 5.85 | 6.08 |
| 36 | R3xL6 | 4.55 | 3.91 | 4.97 | 4.47 | 4.83 |
| 37 | R1xL7 | 4.75 | 3.04 | 5.76 | 4.00 | 6.21 |
| 38 | R2xL7 | 5.30 | 4.21 | 7.07 | 4.49 | 5.41 |
| 39 | S1xL7 | 4.85 | 3.05 | 5.33 | 4.49 | 6.53 |
| 40 | S2xL7 | 4.93 | 3.88 | 5.16 | 5.38 | 5.30 |
| 41 | S3xL7 | 4.96 | 2.95 | 6.22 | 3.89 | 6.79 |
| 42 | R3xL7 | 4.57 | 3.54 | 4.42 | 4.61 | 5.72 |
| 43 | R1xL8 | 5.07 | 3.88 | 4.58 | 6.52 | 5.30 |
| 44 | R2xL8 | 5.85 | 4.89 | 6.30 | 5.07 | 7.14 |
| 45 | S1xL8 | 5.43 | 4.33 | 5.00 | 6.34 | 6.04 |
| 46 | S2xL8 | 5.94 | 4.11 | 5.95 | 6.63 | 7.06 |
| 47 | S3xL8 | 5.88 | 5.00 | 5.76 | 5.14 | 7.61 |
| 48 | R3xL8 | 4.98 | 4.28 | 5.58 | 4.94 | 5.12 |
| 49 | R1xL9 | 5.91 | 4.73 | 5.76 | 6.17 | 6.99 |
| 50 | R2xL9 | 5.52 | 2.98 | 5.92 | 5.69 | 7.47 |
| 51 | S1xL9 | 6.38 | 5.24 | 6.84 | 5.72 | 7.72 |
| 52 | S2xL9 | 6.03 | 4.90 | 6.26 | 4.63 | 8.33 |
| 53 | S3xL9 | 5.90 | 4.32 | 5.81 | 4.99 | 8.46 |
| 54 | R3xL9 | 5.28 | 3.01 | 5.89 | 5.42 | 6.81 |
| 55 | R1xL10 | 4.46 | 3.59 | 5.19 | 2.72 | 6.33 |
| 56 | R2xL10 | 5.38 | 4.29 | 5.24 | 4.95 | 7.06 |
| 57 | S1xL10 | 5.40 | 4.65 | 4.63 | 5.46 | 6.84 |
| 58 | S2xL10 | 5.50 | 4.21 | 6.66 | 5.49 | 5.64 |
| 59 | S3xL10 | 4.54 | 1.78 | 5.73 | 5.15 | 5.51 |
| 60 | R3xL10 | 5.27 | 3.56 | 6.07 | 5.09 | 6.36 |

| | | | | | | |
|---|---|---|---|---|---|---|
| 61 | R1xL11 | 5.09 | 3.86 | 5.52 | 5.24 | 5.75 |
| 62 | R2xL11 | 5.68 | 3.61 | 6.16 | 5.47 | 7.47 |
| 63 | S1xL11 | 5.69 | 5.58 | 4.12 | 5.81 | 7.24 |
| 64 | S2xL11 | 6.07 | 4.24 | 6.11 | 6.78 | 7.15 |
| 65 | S3xL11 | 6.17 | 4.21 | 5.80 | 6.92 | 7.74 |
| 66 | R3xL11 | 5.10 | 3.48 | 5.32 | 5.49 | 6.13 |
| 67 | R1xL12 | 5.10 | 2.65 | 5.90 | 5.17 | 6.68 |
| 68 | R2xL12 | 6.15 | 4.32 | 6.51 | 4.85 | 8.91 |
| 69 | S1xL12 | 5.25 | 3.81 | 5.67 | 5.45 | 6.06 |
| 70 | S2xL12 | 5.54 | 3.91 | 7.41 | 3.44 | 7.39 |
| 71 | S3xL12 | 6.64 | 6.20 | 7.35 | 5.16 | 7.84 |
| 72 | R3xL12 | 5.53 | 3.88 | 6.56 | 5.08 | 6.61 |
| 73 | R1xL13 | 5.00 | 3.90 | 4.63 | 5.16 | 6.33 |
| 74 | R2xL13 | 5.80 | 4.35 | 6.27 | 4.83 | 7.75 |
| 75 | S1xL13 | 5.90 | 4.17 | 6.20 | 5.98 | 7.25 |
| 76 | S2xL13 | 5.49 | 4.68 | 5.33 | 5.07 | 6.88 |
| 77 | S3xL13 | 6.10 | 5.24 | 6.40 | 5.39 | 7.36 |
| 78 | R3xL13 | 4.87 | 3.79 | 4.25 | 5.36 | 6.07 |
| 79 | R1xL14 | 5.66 | 4.60 | 5.73 | 6.02 | 6.28 |
| 80 | R2xL14 | 5.92 | 4.22 | 6.56 | 5.90 | 6.99 |
| 81 | S1xL14 | 5.72 | 3.54 | 6.59 | 5.45 | 7.29 |
| 82 | S2xL14 | 6.10 | 4.59 | 6.54 | 6.82 | 6.44 |
| 83 | S3xL14 | 5.25 | 3.95 | 6.75 | 3.85 | 6.45 |
| 84 | R3xL14 | 4.71 | 3.97 | 5.79 | 4.27 | 4.81 |
| 85 | R1xL15 | 5.56 | 4.64 | 6.35 | 5.24 | 6.02 |
| 86 | R2xL15 | 6.61 | 5.57 | 7.46 | 5.80 | 7.62 |
| 87 | S1xL15 | 5.85 | 3.60 | 7.41 | 6.06 | 6.32 |
| 88 | S2xL15 | 6.05 | 4.36 | 6.72 | 5.74 | 7.38 |
| 89 | S3xL15 | 6.01 | 4.63 | 7.26 | 5.33 | 6.80 |
| 90 | R3xL15 | 5.56 | 4.73 | 7.52 | 4.88 | 5.11 |
| 91 | R1xL16 | 5.21 | 3.94 | 5.53 | 5.43 | 5.96 |
| 92 | R2xL16 | 5.51 | 3.92 | 6.12 | 6.02 | 6.00 |

| | | | | | | |
|---|---|---|---|---|---|---|
| 93 | S1xL16 | 5.78 | 4.33 | 5.97 | 5.81 | 7.01 |
| 94 | S2xL16 | 5.31 | 4.54 | 5.42 | 5.70 | 5.58 |
| 95 | S3xL16 | 5.29 | 3.97 | 5.90 | 5.11 | 6.19 |
| 96 | R3xL16 | 4.43 | 3.99 | 2.97 | 5.23 | 5.53 |
| 97 | R1xL17 | 4.88 | 4.54 | 4.90 | 4.34 | 5.73 |
| 98 | R2xL17 | 6.13 | 3.86 | 7.51 | 5.73 | 7.42 |
| 99 | S1xL17 | 5.90 | 4.56 | 5.08 | 6.01 | 7.97 |
| 100 | S2xL17 | 5.26 | 5.24 | 5.99 | 3.80 | 6.02 |
| 101 | S3xL17 | 5.94 | 4.72 | 6.36 | 5.59 | 7.09 |
| 102 | R3xL17 | 5.22 | 4.30 | 4.65 | 6.37 | 5.58 |
| 103 | R1xL18 | 5.10 | 4.38 | 6.09 | 4.71 | 5.21 |
| 104 | R2xL18 | 5.86 | 4.24 | 6.32 | 5.76 | 7.12 |
| 105 | S1xL18 | 5.05 | 4.02 | 5.31 | 5.04 | 5.84 |
| 106 | S2xL18 | 4.98 | 3.82 | 6.09 | 3.47 | 6.55 |
| 107 | S3xL18 | 5.82 | 5.37 | 5.97 | 4.49 | 7.45 |
| 108 | R3xL18 | 5.28 | 3.60 | 6.36 | 5.35 | 5.80 |
| 109 | R1xL19 | 6.31 | 3.38 | 7.39 | 6.14 | 8.34 |
| 110 | R2xL19 | 6.59 | 3.86 | 7.98 | 6.15 | 8.39 |
| 111 | S1xL19 | 5.66 | 4.27 | 5.54 | 5.56 | 7.29 |
| 112 | S2xL19 | 6.10 | 4.31 | 7.24 | 4.92 | 7.92 |
| 113 | S3xL19 | 5.84 | 4.31 | 5.98 | 5.47 | 7.59 |
| 114 | R3xL19 | 4.87 | 3.97 | 4.92 | 4.53 | 6.06 |
| 115 | R1xL20 | 5.84 | 3.44 | 7.18 | 6.02 | 6.73 |
| 116 | R2xL20 | 6.10 | 3.86 | 6.69 | 6.25 | 7.60 |
| 117 | S1xL20 | 6.05 | 4.67 | 6.14 | 6.60 | 6.78 |
| 118 | S2xL20 | 5.89 | 3.52 | 5.86 | 6.37 | 7.81 |
| 119 | S3xL20 | 6.24 | 4.31 | 7.17 | 6.22 | 7.28 |
| 120 | R3xL20 | 5.19 | 4.30 | 6.00 | 4.65 | 5.83 |

Supplementary Table S7C. Least square means at 4 environments and across-environments for bright greenish yellow fluorescence score (pFLt) for 120 hybrids formed by crossing 6 inbred tester lines (R1-R3 are *A. flavus* resistant, and S1-S3 are susceptible) with 20 inbred maize lines with varying concentrations of carotenoids in grain (L1-L20).

| Hybrid | T x L | Across | AF12 | AF13 | TL12 | TL13 |
|---|---|---|---|---|---|---|
| 1 | R1xL1 | 4.90 | 6.76 | 3.91 | 3.30 | 5.64 |
| 2 | R2xL1 | 4.30 | 5.19 | 3.62 | 3.02 | 5.36 |
| 3 | S1xL1 | 5.19 | 6.36 | 3.54 | 4.79 | 6.08 |
| 4 | S2xL1 | 3.81 | 3.91 | 3.54 | 3.54 | 4.27 |
| 5 | S3xL1 | 4.47 | 6.01 | 3.54 | 3.54 | 4.79 |
| 6 | R3xL1 | 4.27 | 4.27 | 4.27 | 4.27 | 4.27 |
| 7 | R1xL2 | 4.45 | 5.00 | 3.91 | 4.64 | 4.27 |
| 8 | R2xL2 | 4.94 | 6.36 | 3.54 | 4.42 | 5.43 |
| 9 | S1xL2 | 4.70 | 6.07 | 3.54 | 3.54 | 5.64 |
| 10 | S2xL2 | 5.21 | 7.89 | 3.54 | 4.27 | 5.15 |
| 11 | S3xL2 | 4.64 | 5.84 | 4.27 | 3.91 | 4.55 |
| 12 | R3xL2 | 4.64 | 5.20 | 4.27 | 2.78 | 6.32 |
| 13 | R1xL3 | 3.72 | 3.91 | 3.54 | 3.54 | 3.91 |
| 14 | R2xL3 | 4.07 | 4.92 | 3.54 | 3.91 | 3.91 |
| 15 | S1xL3 | 4.18 | 5.00 | 3.54 | 3.91 | 4.27 |
| 16 | S2xL3 | 3.72 | 3.91 | 3.54 | 2.14 | 5.28 |
| 17 | S3xL3 | 4.35 | 5.31 | 3.54 | 3.54 | 5.00 |
| 18 | R3xL3 | 4.05 | 5.20 | 3.54 | 3.54 | 3.91 |
| 19 | R1xL4 | 3.19 | 2.66 | 3.54 | 2.66 | 3.91 |
| 20 | R2xL4 | 4.27 | 4.55 | 3.54 | 4.42 | 4.55 |
| 21 | S1xL4 | 4.34 | 5.28 | 3.54 | 3.54 | 5.00 |
| 22 | S2xL4 | 4.25 | 4.64 | 3.54 | 3.91 | 4.92 |
| 23 | S3xL4 | 4.74 | 5.52 | 5.28 | 3.91 | 4.27 |
| 24 | R3xL4 | 3.91 | 4.64 | 3.91 | 3.54 | 3.54 |
| 25 | R1xL5 | 3.99 | 3.54 | 3.54 | 3.54 | 5.36 |
| 26 | R2xL5 | 4.43 | 5.00 | 3.54 | 4.19 | 5.00 |
| 27 | S1xL5 | 4.30 | 4.92 | 3.54 | 4.19 | 4.55 |
| 28 | S2xL5 | 4.23 | 4.55 | 3.91 | 3.91 | 4.55 |

| | | | | | | |
|---|---|---|---|---|---|---|
| 29 | S3xL5 | 4.63 | 4.42 | 4.64 | 3.91 | 5.55 |
| 30 | R3xL5 | 3.35 | 2.66 | 3.54 | 3.67 | 3.54 |
| 31 | R1xL6 | 4.12 | 5.48 | 3.54 | 3.91 | 3.54 |
| 32 | R2xL6 | 4.43 | 6.01 | 3.91 | 3.54 | 4.27 |
| 33 | S1xL6 | 4.70 | 6.55 | 3.54 | 3.54 | 5.19 |
| 34 | S2xL6 | 4.73 | 7.04 | 3.91 | 3.54 | 4.42 |
| 35 | S3xL6 | 5.03 | 6.76 | 3.91 | 4.27 | 5.19 |
| 36 | R3xL6 | 4.29 | 5.52 | 3.54 | 4.19 | 3.91 |
| 37 | R1xL7 | 3.81 | 2.66 | 3.54 | 3.91 | 5.15 |
| 38 | R2xL7 | 4.17 | 5.84 | 3.91 | 3.02 | 3.91 |
| 39 | S1xL7 | 4.40 | 3.54 | 3.54 | 4.27 | 6.25 |
| 40 | S2xL7 | 4.02 | 3.67 | 3.54 | 4.44 | 4.42 |
| 41 | S3xL7 | 4.15 | 4.31 | 3.91 | 4.19 | 4.19 |
| 42 | R3xL7 | 3.76 | 3.91 | 3.57 | 2.66 | 4.92 |
| 43 | R1xL8 | 4.00 | 5.00 | 3.91 | 3.54 | 3.54 |
| 44 | R2xL8 | 4.80 | 6.32 | 2.66 | 4.19 | 6.04 |
| 45 | S1xL8 | 4.31 | 6.76 | 3.54 | 2.66 | 4.27 |
| 46 | S2xL8 | 4.37 | 5.71 | 3.91 | 2.66 | 5.20 |
| 47 | S3xL8 | 4.75 | 4.92 | 4.92 | 3.91 | 5.28 |
| 48 | R3xL8 | 3.75 | 4.92 | 3.54 | 3.02 | 3.54 |
| 49 | R1xL9 | 4.66 | 5.28 | 3.91 | 3.54 | 5.92 |
| 50 | R2xL9 | 4.96 | 6.37 | 3.91 | 4.19 | 5.38 |
| 51 | S1xL9 | 5.13 | 6.43 | 3.54 | 4.92 | 5.64 |
| 52 | S2xL9 | 5.23 | 7.02 | 3.91 | 3.67 | 6.32 |
| 53 | S3xL9 | 5.07 | 5.28 | 3.91 | 4.19 | 6.92 |
| 54 | R3xL9 | 4.59 | 4.92 | 3.91 | 4.64 | 4.92 |
| 55 | R1xL10 | 3.72 | 4.79 | 3.54 | 2.66 | 3.91 |
| 56 | R2xL10 | 3.68 | 2.66 | 3.54 | 3.91 | 4.63 |
| 57 | S1xL10 | 3.69 | 4.55 | 2.66 | 3.02 | 4.55 |
| 58 | S2xL10 | 4.16 | 4.55 | 3.54 | 4.27 | 4.27 |
| 59 | S3xL10 | 3.19 | 2.14 | 3.54 | 3.54 | 3.54 |
| 60 | R3xL10 | 4.27 | 4.27 | 3.54 | 4.64 | 4.63 |

| | | | | | | |
|---|---|---|---|---|---|---|
| 61 | R1xL11 | 3.32 | 2.66 | 3.54 | 3.54 | 3.54 |
| 62 | R2xL11 | 3.72 | 3.91 | 3.54 | 3.54 | 3.91 |
| 63 | S1xL11 | 4.49 | 6.08 | 3.54 | 3.91 | 4.42 |
| 64 | S2xL11 | 4.01 | 4.83 | 3.02 | 4.27 | 3.91 |
| 65 | S3xL11 | 4.25 | 5.28 | 3.54 | 3.91 | 4.27 |
| 66 | R3xL11 | 3.32 | 3.54 | 3.54 | 2.66 | 3.54 |
| 67 | R1xL12 | 3.50 | 3.91 | 3.54 | 3.91 | 2.66 |
| 68 | R2xL12 | 4.16 | 5.00 | 3.54 | 3.54 | 4.55 |
| 69 | S1xL12 | 3.98 | 4.19 | 3.54 | 3.91 | 4.27 |
| 70 | S2xL12 | 4.48 | 4.92 | 4.27 | 3.91 | 4.83 |
| 71 | S3xL12 | 5.82 | 8.65 | 4.64 | 5.07 | 4.92 |
| 72 | R3xL12 | 3.81 | 3.91 | 3.91 | 3.91 | 3.54 |
| 73 | R1xL13 | 3.89 | 4.92 | 3.54 | 3.54 | 3.57 |
| 74 | R2xL13 | 4.43 | 6.08 | 3.54 | 3.54 | 4.55 |
| 75 | S1xL13 | 4.55 | 4.83 | 5.00 | 3.30 | 5.07 |
| 76 | S2xL13 | 4.71 | 7.85 | 3.54 | 3.54 | 3.91 |
| 77 | S3xL13 | 4.32 | 6.29 | 3.91 | 3.02 | 4.05 |
| 78 | R3xL13 | 3.72 | 3.54 | 3.54 | 3.91 | 3.91 |
| 79 | R1xL14 | 3.58 | 4.27 | 3.54 | 3.02 | 3.48 |
| 80 | R2xL14 | 4.34 | 3.91 | 4.27 | 4.64 | 4.55 |
| 81 | S1xL14 | 3.84 | 3.67 | 4.27 | 2.66 | 4.79 |
| 82 | S2xL14 | 3.88 | 3.91 | 3.54 | 4.55 | 3.54 |
| 83 | S3xL14 | 4.17 | 4.92 | 4.27 | 2.66 | 4.83 |
| 84 | R3xL14 | 3.63 | 3.91 | 3.54 | 3.54 | 3.54 |
| 85 | R1xL15 | 4.17 | 5.28 | 3.54 | 3.02 | 4.83 |
| 86 | R2xL15 | 5.45 | 6.36 | 4.55 | 5.07 | 5.84 |
| 87 | S1xL15 | 4.88 | 5.56 | 3.91 | 5.15 | 4.92 |
| 88 | S2xL15 | 4.95 | 5.43 | 3.91 | 4.19 | 6.29 |
| 89 | S3xL15 | 5.67 | 6.08 | 4.19 | 6.08 | 6.36 |
| 90 | R3xL15 | 4.56 | 5.80 | 4.27 | 4.27 | 3.91 |
| 91 | R1xL16 | 3.44 | 3.54 | 2.66 | 3.30 | 4.27 |
| 92 | R2xL16 | 3.97 | 4.92 | 4.05 | 3.02 | 3.91 |

| | | | | | | |
|---|---|---|---|---|---|---|
| 93 | S1xL16 | 3.68 | 5.00 | 3.54 | 2.66 | 3.54 |
| 94 | S2xL16 | 4.12 | 5.00 | 3.54 | 3.39 | 4.55 |
| 95 | S3xL16 | 3.75 | 4.92 | 3.54 | 2.66 | 3.91 |
| 96 | R3xL16 | 3.72 | 3.91 | 3.54 | 3.91 | 3.54 |
| 97 | R1xL17 | 3.04 | 2.66 | 3.54 | 1.77 | 4.19 |
| 98 | R2xL17 | 3.82 | 3.54 | 3.54 | 3.67 | 4.55 |
| 99 | S1xL17 | 4.62 | 4.83 | 3.54 | 4.92 | 5.20 |
| 100 | S2xL17 | 3.72 | 4.27 | 3.54 | 3.54 | 3.54 |
| 101 | S3xL17 | 4.52 | 4.83 | 3.91 | 4.55 | 4.79 |
| 102 | R3xL17 | 3.67 | 3.91 | 3.71 | 3.54 | 3.54 |
| 103 | R1xL18 | 3.88 | 4.55 | 3.54 | 3.54 | 3.91 |
| 104 | R2xL18 | 4.07 | 5.28 | 3.54 | 3.54 | 3.91 |
| 105 | S1xL18 | 3.60 | 4.53 | 1.77 | 4.19 | 3.91 |
| 106 | S2xL18 | 3.81 | 4.64 | 3.54 | 3.54 | 3.54 |
| 107 | S3xL18 | 4.53 | 6.08 | 3.91 | 3.54 | 4.60 |
| 108 | R3xL18 | 3.72 | 3.91 | 3.91 | 3.54 | 3.54 |
| 109 | R1xL19 | 3.97 | 4.19 | 3.54 | 3.54 | 4.60 |
| 110 | R2xL19 | 4.13 | 4.64 | 3.54 | 3.91 | 4.42 |
| 111 | S1xL19 | 4.88 | 7.28 | 3.54 | 3.91 | 4.79 |
| 112 | S2xL19 | 4.23 | 6.32 | 3.57 | 2.14 | 4.92 |
| 113 | S3xL19 | 4.14 | 6.08 | 3.54 | 3.02 | 3.91 |
| 114 | R3xL19 | 4.13 | 5.15 | 3.91 | 3.91 | 3.54 |
| 115 | R1xL20 | 3.41 | 3.54 | 3.54 | 2.66 | 3.91 |
| 116 | R2xL20 | 4.65 | 5.52 | 3.54 | 4.55 | 5.00 |
| 117 | S1xL20 | 3.81 | 3.91 | 3.54 | 4.27 | 3.54 |
| 118 | S2xL20 | 3.92 | 3.91 | 3.54 | 2.66 | 5.56 |
| 119 | S3xL20 | 4.32 | 4.27 | 3.54 | 3.54 | 5.92 |
| 120 | R3xL20 | 4.07 | 4.92 | 3.91 | 3.91 | 3.54 |

Supplementary Table S8A. Summary analysis of variance for aflatoxin concentration on grain (AFTt) of 120 maize hybrids grown at five environments, considering environment effects as random, and all other effects as described in the Materials and Methods.

| | ProVA | BC | BCX | ZX | LT |
|---|---|---|---|---|---|
| Testers | <.0001 | <.0001 | <.0001 | <.0001 | <.0001 |
| R vs S | <.0001 | <.0001 | <.0001 | <.0001 | <.0001 |
| Lines | <.0001 | <.0001 | <.0001 | <.0001 | <.0001 |
| Hi vs Lo | 0.0123 | <.0001 | 0.0005 | <.0001 | <.0001 |
| (R vs S) x Loc | <.0001 | <.0001 | <.0001 | <.0001 | <.0001 |
| (Hi vs Lo) x Loc | 0.0018 | 0.0282 | 0.0942 | 0.0126 | <.0001 |

Supplementary Table S8B. Summary analysis of variance for ear rot symptom scores (pERt) for 120 maize hybrids grown at four environments, considering environment effects as random, and all other effects as described in the Materials and Methods.

| | ProVA | BC | BCX | ZX | LT |
|---|---|---|---|---|---|
| Testers | <.0001 | <.0001 | <.0001 | <.0001 | <.0001 |
| R vs S | <.0001 | <.0001 | <.0001 | <.0001 | <.0001 |
| Lines | <.0001 | <.0001 | <.0001 | <.0001 | <.0001 |
| Hi vs Lo | <.0001 | <.0001 | <.0627 | <.5020 | <.0001 |
| (R vs S) x Loc | 0.2928 | 0.2928 | 0.2928 | 0.2928 | 0.2928 |
| (Hi vs Lo) x Loc | 0.1358 | 0.0310 | 0.1840 | 0.4479 | 0.0291 |

Supplementary Table S8C. Summary analysis of variance for bright greenish yellow fluorescence scores (pFLt) for 120 maize hybrids grown at four environments, considering environment effects as random, and all other effects as described in the Materials and Methods.

| | ProVA | BC | BCX | ZX | LT |
|---|---|---|---|---|---|
| Testers | <.0001 | <.0001 | <.0001 | <.0001 | <.0001 |
| R vs S | <.0001 | <.0001 | <.0001 | <.0001 | <.0001 |
| Lines | <.0001 | <.0001 | <.0001 | <.0001 | <.0001 |
| Hi vs Lo | 0.1754 | 0.0130 | 0.1366 | 0.0166 | 0.0040 |
| (R vs S) x Loc | <.0001 | <.0001 | <.0001 | <.0001 | <.0001 |
| (Hi vs Lo) x Loc | 0.5522 | 0.0007 | 0.8232 | 0.2604 | 0.6508 |

*ProVA, BC, BCX, ZX, LT: total provitamin A, beta-carotene, beta-cryptoxanthin, zeaxanthin and lutein, respectively. R, S: A. flavus resistant and susceptible tester lines. Hi and Lo: lines with high or low concentrations of the respective carotenoids. Loc, location or environment.*
